# Oxygen-mediated projection microstereolithography for sub–100-μm zirconia dental veneers

**DOI:** 10.1126/sciadv.aef9661

**Published:** 2026-11-04

**Authors:** Yanru Shen, Xiang Wang, Chen Yang, Ying Jiang, Feng Wang, Zhankun Pan, Jinhua Li, Nicholas X. Fang, Yuchun Sun

**Affiliations:** ^1^ Center of Digital Dentistry, Department of Prosthodontics, Peking University School and Hospital of Stomatology; National Center for Stomatology; National Clinical Research Center for Oral Diseases; National Engineering Research Center of Oral Biomaterials and Digital Medical Devices; Beijing Key Laboratory for Intelligent Biomanufacturing and Regeneration of Craniofacial Tissues; NHC Key Laboratory of Digital Stomatology; Key Laboratory of Digital Stomatology, Chinese Academy of Medical Sciences; NMPA Key Laboratory for Dental Materials, Beijing 100081, PR China.; ^2^ BMF Nano Material Technology Co., Ltd, Shenzhen 518017, Guangdong Province, PR China.; ^3^ Department of Mechanical Engineering, University of Hong Kong, Hong Kong, PR China.

## Abstract

High-resolution vat photopolymerization of ceramics is constrained by light scattering, which broadens cured features, and by shallow penetration, which limits interlayer bonding. This trade-off is particularly damaging for microscale structures such as no-preparation dental veneers. We introduce an oxygen-mediated projection microstereolithography strategy in which an oxygen-permeable release film creates an interfacial inhibition field in high-solid–loading zirconia slurry. Rather than uniformly reducing curing, oxygen preferentially filters low-dose scattered light at pattern edges while preserving curing in the projected core. This anisotropic inhibition reshapes the accuracy-depth trade-off, enabling optimized multilayer printing of thin-wall zirconia. The process enabled reproducible fabrication of ∼80-micrometer zirconia veneers with high trueness, improved marginal adaptation, and handling-related strength comparable to substantially thicker milled veneers. The approach provides the potential for minimally invasive dental restoration and a general strategy for high-resolution additive manufacturing of strongly scattering ceramic suspensions.

## INTRODUCTION

Ceramics combine high stiffness, chemical stability, wear resistance, and thermal tolerance, making them indispensable for dental restorations, biomedical implants, microfluidic devices, micro-optics, and structural components operating in harsh environments. Yet, these same properties make ceramics difficult to machine once densified. Digital light-based additive manufacturing, including digital light processing and projection microstereolithography (PμSL) (Fig. 1A), offers a route to fabricate complex ceramic green bodies from particle-loaded photopolymer slurries, followed by debinding and sintering (*1*, *2*). This approach is particularly attractive when the target structure contains thin walls, sharp margins, or high-curvature surfaces that are difficult to obtain by subtractive machining.

**Fig. 1.**
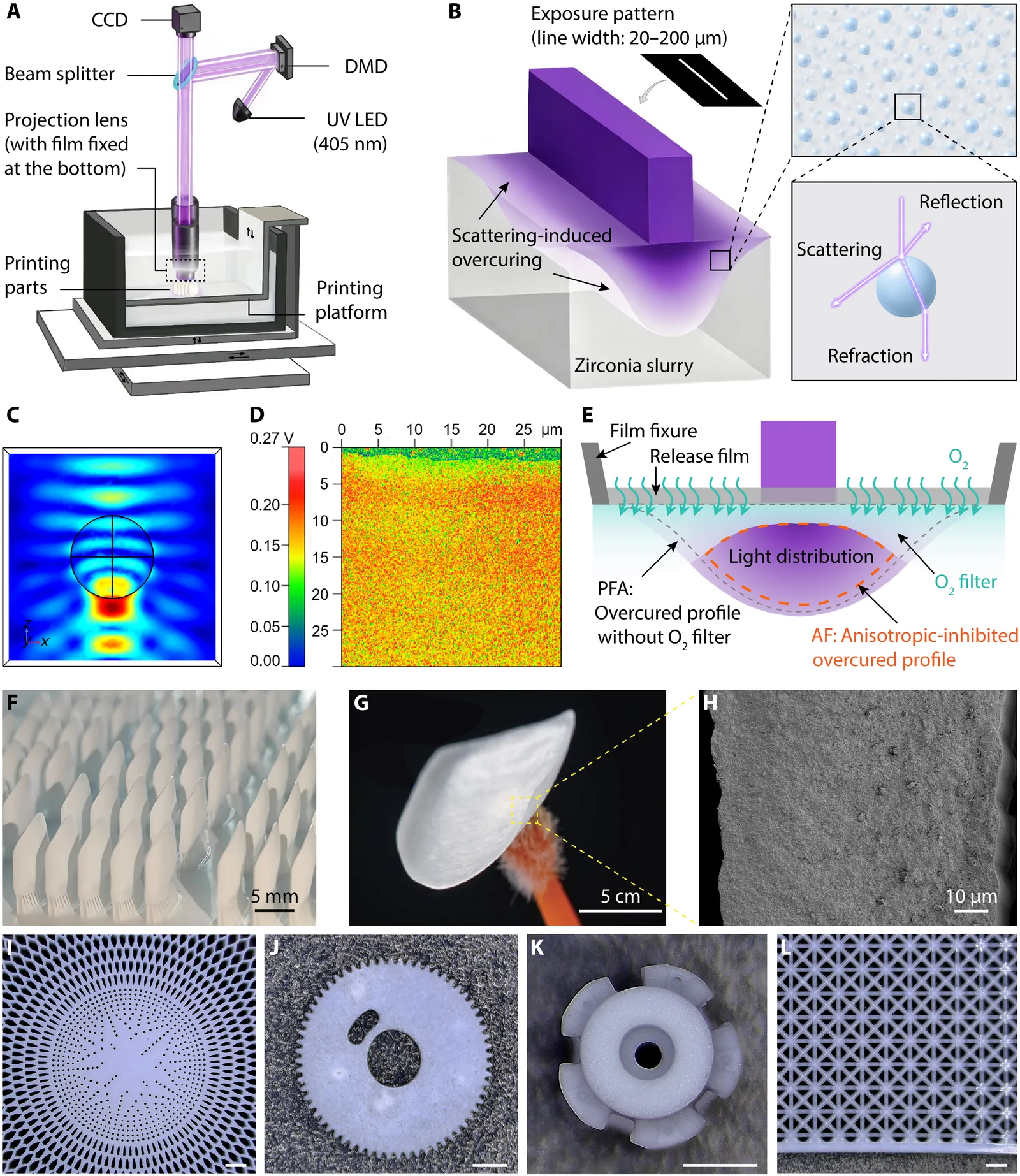
Oxygen-mediated PμSL for high-precision ceramic structures with micrometer-scale features. (**A**) Schematic of the PμSL system setup incorporating an oxygen-permeable release film at the release-film/slurry interface. Graphics were created by S. Chen using Procreate. (**B**) Schematic of the projected light distribution during line exposure in a highly loaded zirconia slurry. Suspended ceramic particles perturb light propagation through scattering, reflection, diffraction, and refraction. Graphics were created by S. Chen using Procreate. (**C**) Simulated optical field around a ceramic particle, illustrating particle-induced redistribution of light and forward propagation within the slurry. (**D**) Nano-infrared chemical mapping of the oxygen-inhibited region beneath the oxygen-permeable film. (**E**) Proposed oxygen-mediated threshold-filtering mechanism: In the absence of interfacial oxygen inhibition, scattered light can induce lateral overcuring. Oxygen supplied through the permeable film preferentially suppresses polymerization driven by weak scattered light, while the high-dose projected core remains curable. This steepens the effective curing gradient and improves lateral feature definition. Graphics were created by S. Chen using Procreate. (**F** to **H**) Sub–100-μm zirconia veneers fabricated by oxygen-mediated PμSL: (F) printed veneer green bodies, (G) sintered ultrathin zirconia veneer, and (H) scanning electron microscopy (SEM) image of the veneer margin showing an edge thickness of ∼80 μm. (**I** to **L**) Representative alumina microstructures fabricated by oxygen-mediated PμSL, illustrating the generality of this strategy to other highly scattering ceramics with complex positive and negative features (scale bar, 1 mm).

Dental veneers provide a demanding clinical example of this manufacturing challenge (*3*, *4*). Veneers are used to address a range of esthetic and functional concerns, including discoloration, malocclusion, and diastema, while preserving as much healthy enamel as possible. They must simultaneously satisfy esthetic appearance, marginal accuracy, bonding reliability, and fracture resistance (*5*–*8*). Conventional heat-pressed or computer numerical control (CNC)–milled glass-ceramic veneers generally require thicknesses on the order of several hundred micrometers to avoid chipping during milling, handling, and service (*9*–*12*). Reducing veneer thickness to the sub–100-μm scale could shift the clinical workflow toward no-preparation or minimally invasive restoration (*13*–*19*), but such geometries are beyond the practical accuracy and damage tolerance of most ceramic manufacturing processes (*20*–*22*). Recent advances in ceramic additive manufacturing have demonstrated the feasibility of fabricating dental ceramic restorations with improved geometric freedom and clinically relevant accuracy, although achieving reliable high-resolution fabrication remains challenging for highly scattering ceramic suspensions (*12*, *18*, *23*). Zirconia, especially 3–mol % yttria-stabilized tetragonal zirconia polycrystal (3YSZ), is mechanically attractive for this purpose, but its high refractive index and strong light scattering create a major barrier for high-resolution photopolymerization (*24*–*27*).

In ceramic vat photopolymerization, suspended high–refractive index particles laterally redistribute photons and create a low-intensity scattering tail that can exceed the local gelation threshold outside the intended feature (Fig. 1B). This scattering-induced overcuring is particularly damaging for microscale features. When the target width is only tens of micrometers, even a small absolute broadening represents a large fractional error. Simply lowering the exposure dose or applying grayscale projection compensation is not satisfactory because the dose reduction also decreases curing depth, compromising layer-to-layer bonding and the integrity of fragile thin-wall green bodies (*28*). Thus, ceramic PμSL faces a coupled trade-off between lateral accuracy and vertical bonding that is not captured by conventional one-dimensional working-curve descriptions.

Oxygen inhibition offers a possible way to reshape this trade-off. In continuous liquid interface production (CLIP), an oxygen-permeable window generates a liquid dead zone that suppresses adhesion and enables continuous replenishment in homogeneous photopolymer resins (*29*, *30*). High-solid-loading ceramic slurries, however, are fundamentally different from transparent resins. The optical field is spatially heterogeneous, the radical generation rate varies sharply between the projected core and the scattered periphery, and oxygen transport occurs through a tortuous resin phase constrained by densely packed particles (*31*, *32*). Optical simulation around a single ceramic particle shows that particles redistribute the incident field (Fig. 1C). Although lateral scattering is generated, a substantial forward-biased component is retained, consistent with Mie scattering (*33*). These features suggest that oxygen inhibition in ceramic PμSL may not act as a uniform offset. Instead, if oxygen preferentially quenches radicals generated by weak scattered light while allowing the high-dose projected core to reach gelation, it could function as a spatially selective chemical filter for the scattering tail. The oxygen-inhibited interfacial region observed beneath the oxygen-permeable film supports the feasibility of such interfacial regulation (Fig. 1D).

Therefore, we introduced an oxygen-mediated PμSL strategy for high-precision printing of ultrathin zirconia veneers and other strongly scattering ceramic microstructures. Building upon CLIP technology and oxygen inhibition, we investigate how an oxygen-supplied interface interacts with the spatially heterogeneous scattering field of a highly loaded ceramic suspension. By integrating an oxygen-permeable amorphous fluoropolymer (AF) release film into the projection-window/slurry interface, we established an interfacial inhibition field that coupled with particle-induced multiple scattering. We termed this effect oxygen-mediated anisotropic inhibition, whereby low-dose scattering-induced polymerization at the lateral periphery was preferentially suppressed, while ensuring the retention of sufficient curing depth in the high-dose projected core (Fig. 1E).

We combined single-line curing experiments, cross-sectional imaging, and a reduced optical-threshold contour model to show that this mechanism reshaped the curing window rather than merely reducing the overall exposure. We then translated the mechanism into a multilayer process window that balanced accuracy, green-body strength, and throughput, followed by thermal processing to dense 3YSZ. Using this strategy, zirconia veneers with a target thickness of ∼80 μm were reproducibly fabricated and sintered, demonstrating a notable reduction in thickness while exhibiting enhanced trueness and marginal adaptation, and handling-related strength that is comparable to conventional milled veneers (Fig. 1, F to H). Beyond dentistry, the same interfacial photochemical principle is applicable to other highly scattering ceramic systems, as demonstrated by high-resolution alumina microstructures with complex positive and negative features (Fig. 1, I to L). Oxygen-mediated PμSL therefore provides not only a manufacturing route for minimally invasive zirconia veneers but also a general framework for controlling light-based printing in strongly scattering particle-filled photopolymers.

## RESULTS

### Oxygen-mediated anisotropic inhibition in scattering ceramic PμSL

The ultrathin veneer geometry is an irregular thin shell with a designed wall thickness mainly ranging from 20 to 200 μm. When the tooth long axis is oriented perpendicular to the build platform, the sliced projection patterns are dominated by fine curved line features whose local width is determined by veneer thickness and surface curvature (Fig. 2A). We therefore used line patterns as simplified single-layer models to investigate the curing behavior of veneer-like edge features. To examine the role of oxygen permeability at the curing interface, two commercial release films were compared: an oxygen-impermeable perfluoroalkoxy alkane (PFA) film and an oxygen-permeable AF film. Under identical 80-μm-width projection and a fixed intensity of 50 mW/cm^2^, the exposure time was varied from 2 to 20 s, corresponding to an exposure dose of 100 to 1000 mJ/cm^2^. Representative white-light interferometric profiles showed that the PFA film produced a broad, film-attached cured region with pronounced lateral spreading, whereas the AF film produced a much narrower cured profile with a sharper lateral boundary (Fig. 2B).

**Fig. 2.**
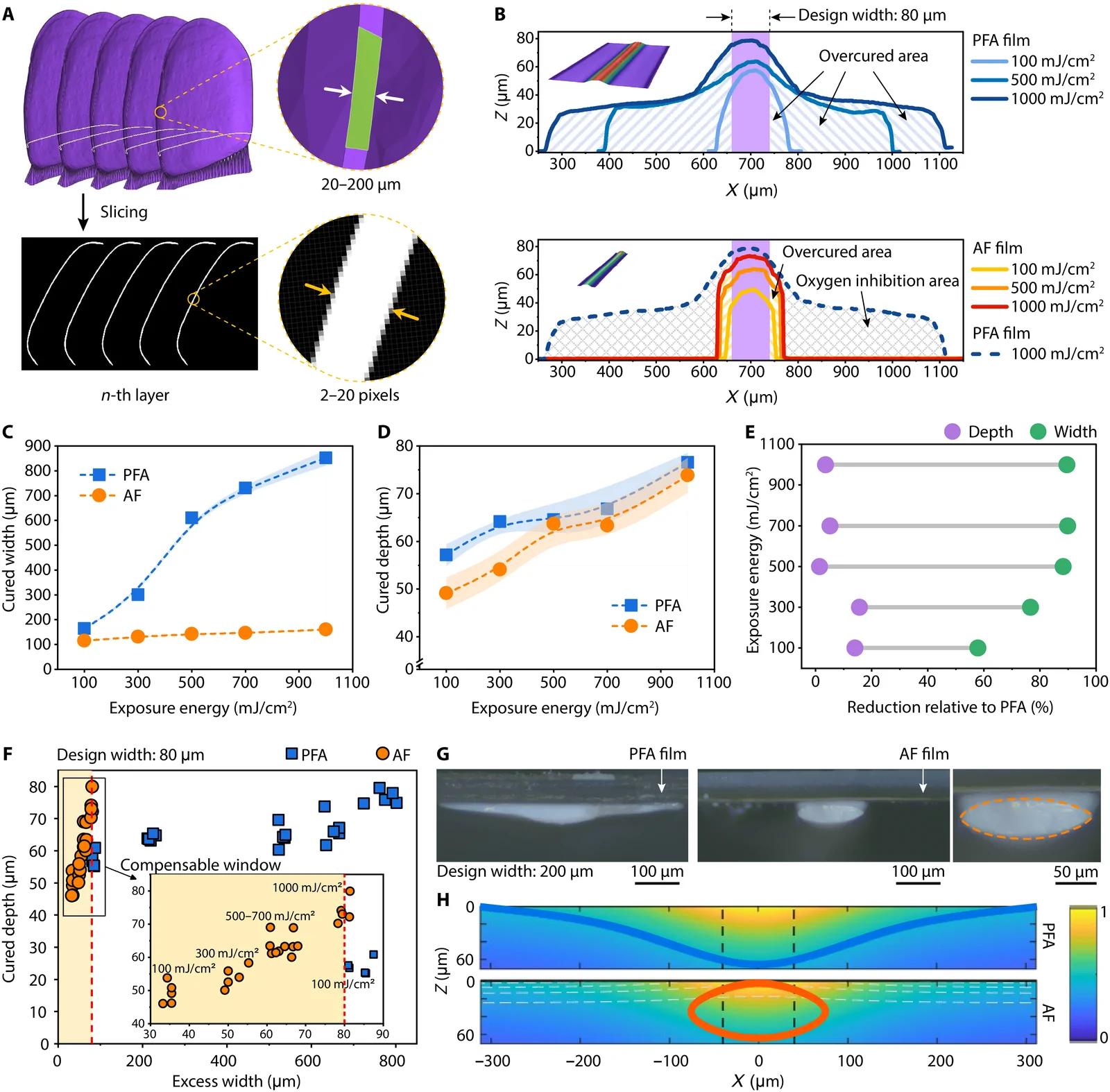
Oxygen-mediated anisotropic inhibition in ceramic PμSL. (**A**) Ultrathin zirconia veneer geometry with wall thickness of 20 to 200 μm and corresponding sliced projection patterns with 2 to 20 pixels wide. (**B**) Representative curing profiles of 80-μm line features fabricated using oxygen-impermeable PFA and oxygen-permeable AF release films under different exposure dose (*I* = 50 mW/cm^2^). (**C**) AF film strongly limited lateral broadening compared with PFA (mean ± SD, *n* = 5). (**D**) AF film only modestly reduced cured depth compared with PFA (mean ± SD, *n* = 5). (**E**) Width reduction when replacing PFA with AF was consistently greater than the depth reduction, indicating anisotropic inhibition. (**F**) Curing-window map plotting excess width against cured depth. The shaded region denotes a compensation-accessible window for an 80-μm target feature. (**G**) Representative optical cross sections of cured specimens fabricated using PFA and AF films. (**H**) Optical-threshold contour model. PFA was simulated using a constant curing threshold, whereas AF was simulated using a near-interface oxygen-shifted threshold with stronger effective inhibition in low-dose regions.

Quantitative analysis confirmed this disproportionate response. As the exposure dose increased, the cured width increased rapidly in the PFA group, indicating severe scattering-induced lateral overcuring (*24*). In contrast, the AF group maintained a smaller cured width closer to the design width across the same exposure range (Fig. 2C). Notably, this improvement in lateral accuracy was not accompanied by a comparable loss of cured depth (Fig. 2D). Excess width is defined as the difference between the cured width and the design width. At the practical exposure dose of 500 mJ/cm^2^, the AF film reduced the excess width by 88.3% relative to PFA, while the cured depth decreased by only 1.4%. Across all tested exposure doses, the reduction in excess width was markedly greater than the reduction in cured depth (Fig. 2E), demonstrating that oxygen permeation did not simply weaken polymerization globally. Instead, it preferentially suppressed the scattering-induced lateral curing component. The consequence of this anisotropic inhibition is more clearly visualized in a curing-window map, where excess width was plotted against cured depth (Fig. 2F). An excess width below the 80-μm target width was used as a first-order compensation-accessible criterion, because larger overcuring would require a negative compensated width. Under these criteria, most AF conditions fell within the compensable printing window, whereas the PFA condition exceeded the lateral compensation limit even at the lowest tested dose. These results indicate that the AF interface shifts the curing trajectory from a scattering-dominated regime to a precision-accessible regime.

The optical cross-sectional morphology of the cured samples provides further support for the proposed oxygen-mediated threshold filtering mechanism (Fig. 2G). The PFA samples exhibit a broad film-attached overcured area, whereas the AF samples display a narrower, lens-shaped cured region separated from the film by a nonuniform inhibition zone.

To rationalize the observed cross-sectional morphology, we constructed a reduced two-dimensional optical-threshold contour model. The model described how a scattering-broadened optical dose field interacts with an effective oxygen-shifted curing threshold. The projected optical dose was described as a scattering broadened field attenuated along the depth direction

E(x,z)=E0 [M(x)⊗K(x)] e−μz (1)

where $E_{0}$ is proportional to the incident exposure dose, M(x) is the projected line mask, K(x) is an effective lateral spreading kernel and a long-tail scattering component, and μ is the effective attenuation coefficient. For a projected line feature, the design mask was defined as

$$M\left(x\right)=\left\{ \begin{array}{cc} 1, & |x|\leq \frac{W_{d}}{2}\\ 0, & |x|>\frac{W_{d}}{2} \end{array}\right.$$ (2)

where $W_{d}$ is the designed linewidth. The spreading kernel was written as the sum of a narrow projection component and a long-tail scattering component

$$K\left(x\right)=\left(1-\beta \right)\frac{1}{\sqrt{2\pi }\sigma }\text{exp}\left(-\frac{x^{2}}{2\sigma^{2}}\right)+\beta \frac{1}{2L_{s}}\text{exp}\left(-\frac{|x|}{L_{s}}\right)$$ (3)

where σ represents effective near-interface broadening, $L_{s}$ represents the characteristic length of the effective scattering tail, and β is the weighting factor of the long-tail component. The Gaussian term represents the directly projected core, whereas the exponential term represents the long-tail lateral photon redistribution caused by ceramic-particle scattering.

For the oxygen-impermeable PFA film, oxygen replenishment at the curing interface was assumed to be negligible over the exposure period. The cured region was therefore defined by a constant threshold criterion

$$E\left(x,z\right)\geq E_{c}$$ (4)

where $E_{c}$ is the effective critical dose required for gelation. For the AF condition, oxygen supplied through the permeable window was incorporated as a near-interface elevation of the curing threshold

$$E\left(x,z\right)\geq E_{c}+\Delta E_{O_{2}}\left(x,z\right)$$ (5)

where $\Delta E_{O_{2}}\left(x,z\right)$ is the equivalent oxygen-inhibition threshold penalty. Because oxygen-mediated radical quenching is more effective in weakly illuminated regions than in the high-dose projected core, the oxygen-related threshold penalty was written as a dose-dependent inhibition term

$$\Delta E_{O_{2}}\left(x,z\right)=Ae^{-z/\lambda }\left\{1+\eta \left[1-\tilde{E}\left(x,z\right)\right]\right\}$$ (6)

$$\tilde{E}\left(x,z\right)=\frac{E\left(x,z\right)}{\text{max}\left[E\left(x,z\right)\right]}$$ (7)

where A is the effective interfacial inhibition strength, λ is the apparent inhibition length, η represents stronger inhibition in low-dose regions, and $\tilde{E}$ is the normalized local dose.

This model assumes that particle-induced light redistribution can be represented by an effective lateral spreading kernel and depth-dependent attenuation, while oxygen inhibition can be incorporated as an effective near-interface, dose-dependent elevation of the curing threshold. Therefore, it visualizes how an oxygen-shifted threshold reshapes the curing boundary in a scattering ceramic slurry, rather than a quantitative reaction-diffusion model that explicitly resolves local oxygen concentration, radical density, oxygen transport, or detailed polymerization kinetics. The model produces a nonuniform inhibition zone in the AF group. Above the high-dose projected core, the oxygen penalty is partially overcome by strong radical generation, whereas near the lateral scattering tail, the weaker dose cannot efficiently overcome oxygen quenching. Therefore, the simulated AF contour exhibits a thinner dead zone above the center and a thicker inhibition region near the edges, which is consistent with experimental observations (Fig. 2H). Parameter sensitivity analysis confirmed that the simulated contour transition was not specific to a single parameter set (table S1 and figs. S1 to S3).

This agreement supports the proposed oxygen-mediated anisotropic inhibition mechanism: Oxygen preferentially suppresses low-dose scattered photons at the lateral periphery while allowing the high-dose projected core to remain sufficiently cured. The mechanism revealed here differs from the conventional role of oxygen inhibition in CLIP. In resin CLIP, the oxygen-permeable window primarily establishes a liquid dead zone that reduces adhesion and enables continuous replenishment (*29*, *30*). In the present ceramic PμSL system, the dominant limitation is instead the lateral redistribution of photons by high–refractive index zirconia particles. The AF film converts this uncontrolled scattering tail into a chemically filtered boundary: Oxygen raises the local gelation threshold near the interface, and the effect is strongest where radical generation is weak. Therefore, the lateral scattering tail is truncated, while the high-dose projected core remains capable of overcoming oxygen inhibition. This mechanism explains the experimentally observed anisotropic response. The contour simulation and cross-sectional observations further show that the AF inhibition field does not equivalently subtract a uniform planar dead zone but selectively suppresses low-dose scattered light.

### Process-window optimization for multilayer thin-wall zirconia printing

Having established that the AF interface selectively suppresses scattering-induced lateral overcuring while preserving sufficient cured depth, we optimized the printing parameters under the AF condition to translate oxygen-mediated anisotropic inhibition into a practical process window. The single-layer exposure pattern of 80-μm lines under AF-mediated curing confirmed that both excess width and cured depth increased with exposure dose, whether the dose was increased by prolonging exposure time or by increasing intensity (fig. S4). This behavior is consistent with working-curve and ceramic-suspension curing descriptions in which the cured profile is governed by the local optical dose and by scattering/absorption in the slurry (*24*, *34*).

To decouple the effects of intensity and time under nominally identical energy doses, we compared curing outcomes at different I×t combinations with matched E, while explicitly considering the effect of design width (Fig. 3A). Typical cured morphologies and scanning profiles are shown in fig. S5. The excess width is summarized in Fig. 3B. In traditional photocuring models, the cured profile is often treated primarily as a function of radiant exposure (E=I×t), with feature size considered mainly as a geometric input (*34*, *35*). However, our experimental data reveal a substantial size effect in zirconia slurries. Excess width increased with design width, and at a fixed E, higher intensity produced larger excess width; this intensity-dependent difference became more pronounced as design width increased. This behavior can be rationalized using the optical model. Because of the long-tail scattering component, photons generated within the projected core can be laterally redistributed toward the feature boundaries through multiple scattering events. As the design dimension increases, this cumulative effect of scattered photons notably raises, thereby increasing excess width (*36*). While previous studies on macro-scale widths (0.5 to 20 mm) have suggested that broadening tends to plateau (*37*), our results indicate that within the microscale range (0.02 to 0.2 mm), this accumulation effect has not yet saturated. Since the same absolute overcuring represents a much larger fractional error for ultrasmall features, this size effect therefore becomes a dominant constraint when defining process windows for delicate microstructures. This finding suggests that static compensation based only on global exposure dose is insufficient for high-precision zirconia microfabrication and that size-dependent scaling or deconvolution of slice images may be needed.

**Fig. 3.**
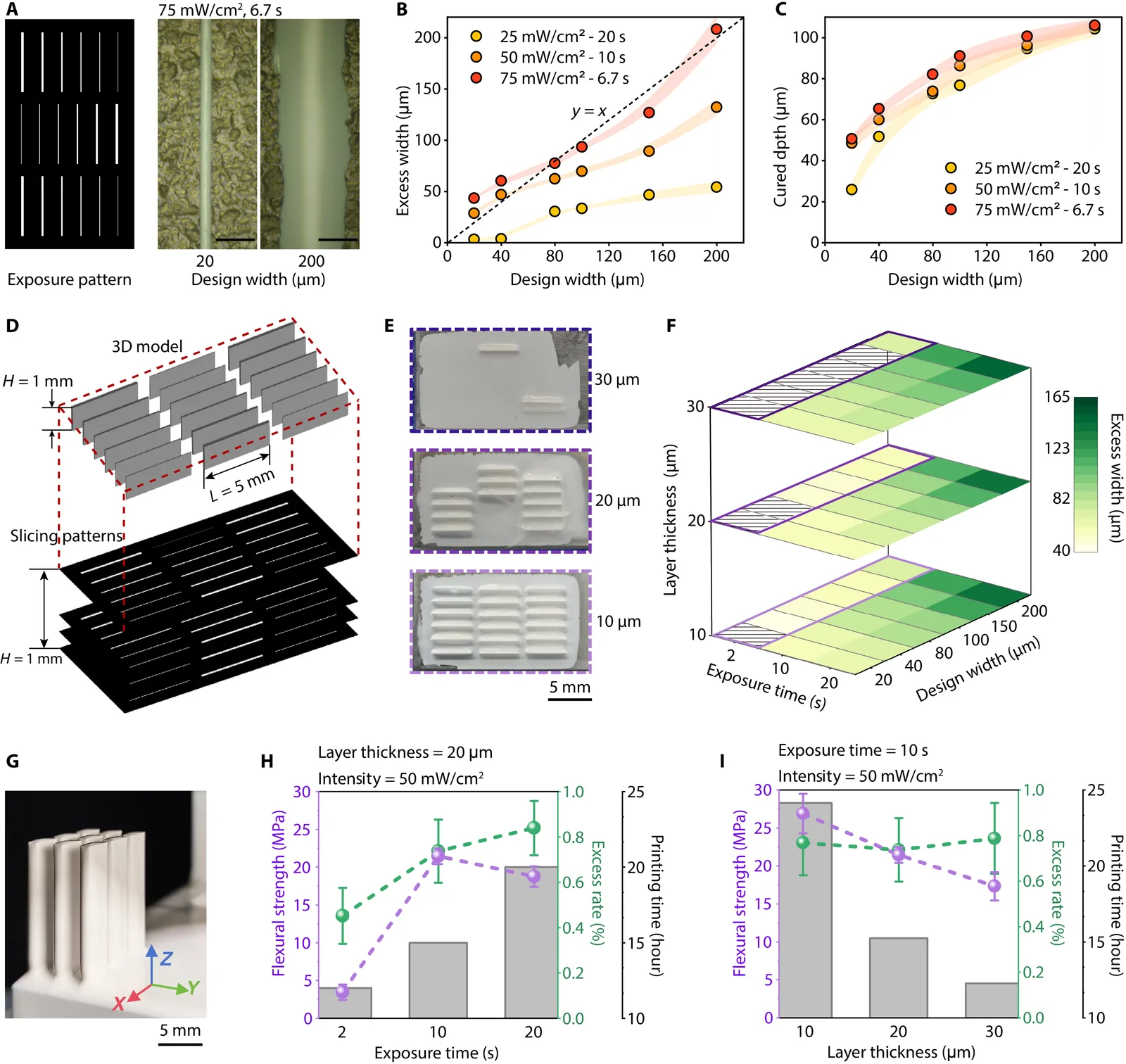
Process-window optimization for thin-wall zirconia: Balancing build integrity, accuracy, and throughput. (**A**) Line pattern with different width (20 to 200 μm) and representative cured lines (scale bar, 100 μm). (**B** and **C**) Feature size–dependent curing using different intensity-time combinations with identical exposure energy and AF film (mean ± SD, *n* = 5). (**D**) Thin-wall models and (**E**) representative printed green-body specimens under different layer thickness. (**F**) Parameter map (heatmap) of excess width across the tested exposure conditions; gray denotes build failure due to insufficient interlayer bonding (mean ± SD, *n* = 5). (**G**) Green-body specimens prepared for flexural strength testing. (**H** and **I**) Multiobjective evaluation of flexural strength, excess rate (ratio of excess width to design width), and total printing time as a function of (H) exposure times and (I) layer thicknesses, revealing an operating window that provides the best trade-off between strength, dimensional accuracy, and fabrication efficiency (mean ± SD, *n* = 5).

Cured depth also increased with design width (Fig. 3C), again highlighting that microscale components do not necessarily follow trends reported for larger geometries. Prior studies suggest that cured depth becomes insensitive to design width once exposure width exceeds ∼1 mm; however, our results show that, below 200 μm, design width can measurably influence depth, consistent with width-dependent light redistribution in scattering slurries. Notably, statistical analysis showed no notable difference in cured depth between 75 mW/cm^2^ for 6.7 s and 50 mW/cm^2^ for 10 s. However, for the 20-μm line exposed at 25 mW/cm^2^ for 20 s, the cured depth was only 25.8 ± 3.6 μm, which is generally insufficient for reliable interlayer bonding in layer-by-layer printing. This indicates that the reciprocity law may not hold under low light-intensity conditions, where reduced radical generation rates in deeper regions cannot overcome chain termination and competing side reactions after substantial attenuation (*38*, *39*). As a result, extending exposure time cannot fully compensate for insufficient intensity, manifesting as a suppressed cured depth.

Together, high-intensity exposure promotes scattering-driven overcuring (detrimental to lateral accuracy), whereas low-intensity exposure risks inadequate depth (detrimental to interlayer bonding and mechanical integrity). Within the parameter space explored here, a moderate exposure intensity (∼50 mW/cm^2^) provides a more robust compromise, enabling adequate cured depth while limiting overcuring, and is therefore better suited for printing ultrathin veneers across a broader thickness range.

Translating the optimized single-layer conditions to three-dimensional veneer-like architectures introduces additional constraints arising from interlayer bonding, cumulative exposure, and the increased sensitivity of thin walls to environmental and process fluctuations. Thin-wall models and representative printed green bodies are shown in Fig. 3 (D and E). When the layer thickness was set to 30 μm, only the 200- and 150-μm sheets could be formed sporadically, and thinner sheets frequently detached during postprinting cleaning. This failure mode indicates insufficient interlayer bonding, consistent with an overly large layer thickness relative to the attainable cured depth and effective interpenetration between layers. A parameter sweep mapped excess width and build success across the tested conditions (Fig. 3F). Gray regions denote printing failure, defined as the inability to form an intact sheet. Within this landscape, a 20-μm layer thickness combined with an exposure time of 10 s produced the most reliable prints while minimizing overcuring, establishing a practical operating window for thin-wall structures. To directly evaluate how photocuring conditions influence structural integrity, we measured the flexural strength of the green bodies using three-point bending (Fig. 3G and fig. S6, A and B), rather than focusing only on sintered specimens. Although sintered strength reflects the final material performance, it convolves photocuring-induced defects with heat treatment–induced densification, shrinkage, and microstructural evolution, limiting its utility for process optimization.

Multiobjective analyses were performed integrating strength, accuracy, and total printing time. At a fixed exposure time, increasing light intensity first improved the green-body flexural strength and then reduced it, with the maximum strength obtained at 50 mW/cm^2^ (fig. S6C). Increasing exposure time inevitably increased total printing time, indicating lower throughput, and enlarged excess width, indicating lower accuracy, while flexural strength showed a similar rise-then-fall behavior. As exposure time of 10 s provided best compromise, yielding peak mechanical performance with moderate accuracy loss and acceptable efficiency (Fig. 3H). These results indicate that green-body strength is not governed simply by maximizing exposure energy. Insufficient exposure results in incomplete polymerization and weak interlayer cohesion, whereas excessive exposure amplifies scattering-induced lateral overcuring and curing heterogeneity. Overcured peripheral regions are mechanically fragile and may act as defect sources during release, recoating, or subsequent layer formation. Prolonged exposure may also increase local conversion gradients and polymerization-shrinkage mismatch, further contributing to internal defects. Therefore, an intermediate exposure condition is required to balance conversion, interlayer bonding, and defect suppression.

Layer thickness introduced an additional trade-off between structural integrity and fabrication efficiency. Reducing the layer thickness increased the ratio of curing depth to deposited layer thickness, thereby improving interlayer overlap and green-body strength (fig. S6D). However, total printing time increased markedly because of the larger number of layers and repeated release/recoating cycles (Fig. 3I). Excessively thin layers may also increase cumulative exposure and process-related defect opportunities without proportionally improving mechanical performance. Considering lateral excess rate, green-body strength and printing time together, 50 mW/cm^2^, 10-s exposure, and 20-μm layer thickness provided the most balanced operating condition. This parameter set preserved sufficient interlayer bonding, limited lateral overcuring and maintained printing efficiency, and was therefore selected for subsequent ultrathin zirconia veneer fabrication.

### Densification and durability of PμSL-fabricated 3YSZ

After defining the oxygen-mediated process window for multilayer thin-wall printing, we evaluated whether the printed green bodies could be converted into dense 3YSZ while retaining dimensional predictability. Thermal analysis revealed the staged decomposition and shrinkage behavior of the photocurable zirconia slurry (Fig. 4A and fig. S7), and the resulting thermal postprocessing yielded intact sintered specimens with comparable linear shrinkage along the *x*, *y*, and *z* directions (23.7 ± 0.1%, 24.0 ± 0.1%, and 23.4 ± 0.1%, respectively; Fig. 4B). Using this schedule, the printed green bodies were converted into sintered zirconia specimens with controlled dimensional shrinkage. This near-isotropic shrinkage indicates that the sintered PμSL-fabricated 3YSZ can be compensated in the digital design with a predictable scaling strategy.

**Fig. 4.**
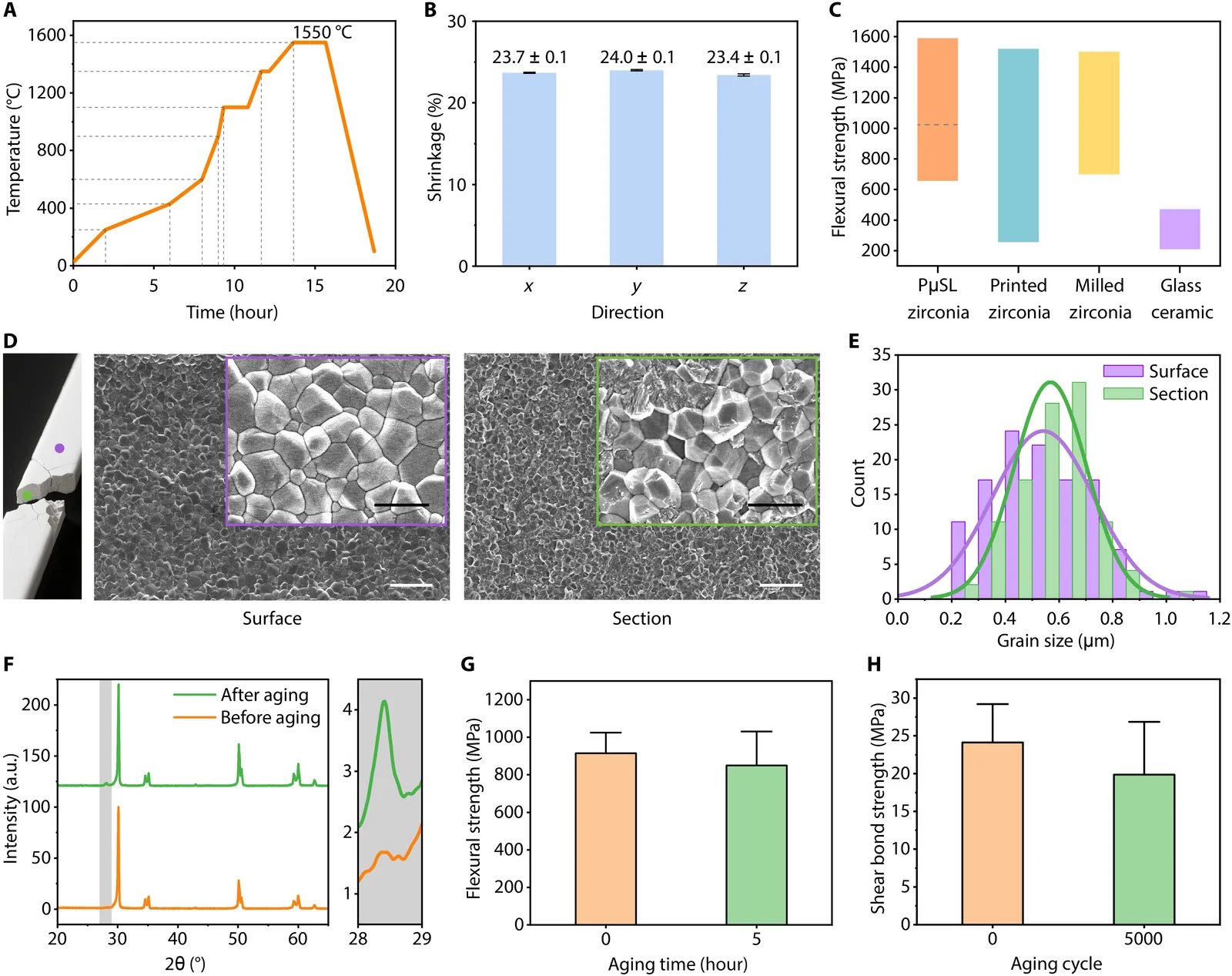
Thermal postprocessing and characterization of PμSL-fabricated zirconia. (**A**) Optimized debinding-sintering schedule derived from thermal analysis. (**B**) Linear shrinkage after sintering along the *x*, *y*, and *z* directions. (**C**) Flexural strength comparison among dental ceramics: PμSL-fabricated 3YSZ in this work (ISO-standard specimens below the dashed line and 80-μm-thick specimens above the dashed line), reported 3D-printing 3YSZ, milled 3YSZ (*41*), and lithium disilicate glass-ceramic (*40*). (**D**) FE-SEM images of the sintered surface and cross section. Scale bars, 1 μm (black) and 3 μm (white). (**E**) Grain-size distribution of the sintered surface and cross section. (**F**) XRD patterns before and after low-temperature aging. The enlarged region highlights the limited monoclinic peak development near 28.1° after aging. (**G**) Flexural strength before and after low-temperature aging (*P* = 0.274, *n* = 9). (**H**) Shear bond strength before and after thermocycling (*P* = 0.018, *n* = 10).

The sintered specimens exhibited high mechanical performance. The flexural strength of ISO-standard specimens reached 915.1 ± 118.9 MPa (Fig. 4C), substantially exceeding that of lithium disilicate glass-ceramic that is commonly used for veneers, and matching values reported for additive-manufactured and milled zirconia (*40*–*42*). In addition, tests were conducted on 80-μm-thick specimens, and these ultrathin specimens exhibited a higher apparent flexural strength. Because brittle-ceramic strength is sensitive to stressed volume and flaw population, this value should be interpreted as geometry-specific evidence of handling reliability rather than as a direct replacement for ISO-standard flexural strength (ISO 6872).

Microstructural analysis further supported the formation of dense and homogeneous zirconia. field-emission scanning electron microscopy images of the sintered surface and cross section showed compact microstructures without obvious interlayer cracks, large pores or lamination defects (Fig. 4D). Grain-size analysis showed similar distributions at the surface and cross section, with mean grain sizes of 0.54 ± 0.18 μm and 0.57 ± 0.17 μm, respectively (Fig. 4E). These results suggest that the printed and sintered ceramic underwent uniform densification through the thickness.

Because 3YSZ can undergo hydrothermal low-temperature degradation through tetragonal-to-monoclinic transformation, we next assessed its phase stability after accelerated aging (ISO 13356). X-ray diffraction (XRD) patterns showed that tetragonal zirconia remained the dominant crystalline phase after aging, with the main peak located near 30.1° (Fig. 4F). A weak monoclinic peak near 28.1°after aging, and local peak area analysis estimated the apparent monoclinic volume fraction to increase from ∼1.3% before aging to ∼4.9% after aging (table S2). Thus, low-temperature aging induced only limited phase transformation under the applied condition. Consistently, flexural strength after aging was 850.2 ± 124.5 MPa, which was not significantly different from the pre-aging strength (*P* = 0.274; Fig. 4G). These results indicate that the limited aging-induced phase transformation did not substantially compromise the mechanical integrity of the sintered PμSL-fabricated 3YSZ.

Last, we evaluated the bonding durability of the sintered 3YSZ under a clinically relevant thermal challenge. Underwent surface glass deposition and selected bonding protocol, shear bond strength decreased from 24.13 ± 2.92 MPa to 19.87 ± 4.28 MPa after thermocycling (*P* = 0.018), but the specimens retained substantial bond strength (Fig. 4H). Collectively, the combination of optimized printing parameters and thermal processing enables PμSL-fabricated 3YSZ to achieve a dense microstructure, stable flexural strength, and long-term bonding strength suitable for ultrathin restorative applications.

### Functional performance of ultrathin PμSL zirconia veneers

Consequently, the reliable fabrication of ultrathin veneers necessitated the integration of oxygen-mediated interfacial regulation, optimized exposure parameters, and controlled thermal processing (fig. S8). On this basis, zirconia veneers with a target thickness of ∼80 μm could be printed and sintered reproducibly (movie S1 and fig. S9A). This thickness is comparable to that of a sheet of A4 paper (∼100 μm) and is notably thinner than traditional CNC-milled veneers (Fig. 5A and fig. S9B). We evaluated the PμSL-fabricated veneers in terms of fabrication quality, esthetic appearance, marginal and internal adaptation, and handling-related mechanical robustness, to determine whether the oxygen-mediated process could be translated from single-feature and thin-wall models to clinically relevant ultrathin dental restorations. Microscopy showed that the veneer margins were continuous and intact, with a uniform thickness and high surface integrity. By contrast, the CNC-milled veneers were substantially thicker and exhibited more frequent marginal defects, consistent with chipping induced by stress concentration during subtractive machining (*43*, *44*). The ultrathin PμSL veneer seated on the corresponding tooth model without an apparent increase in bulk (Fig. 5B), which is beneficial for long-term oral health.

**Fig. 5.**
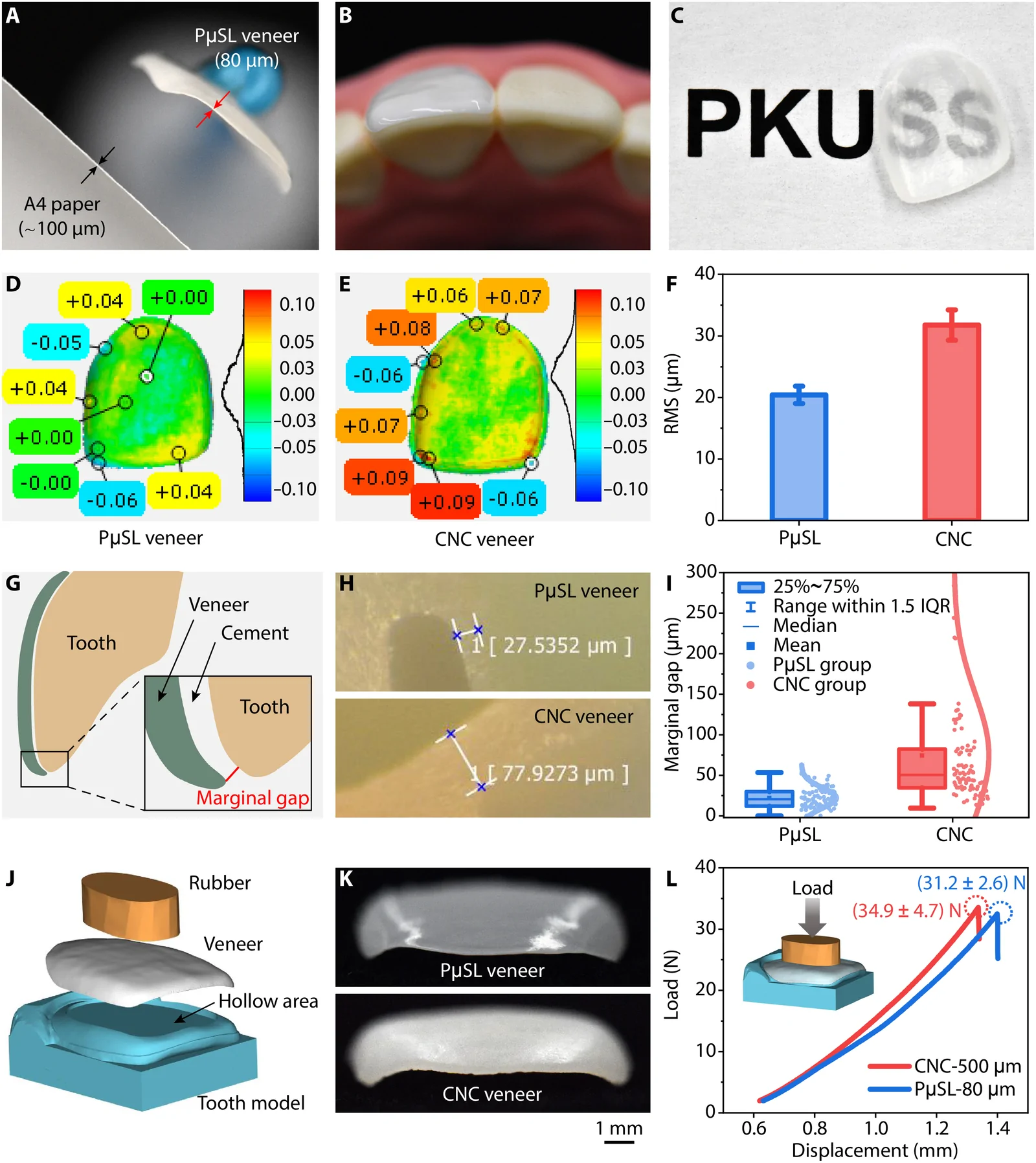
Esthetics, dimensional accuracy, and mechanical performance of PμSL zirconia veneers. (**A** to **C**) Representative printed ultrathin veneer demonstrating (A) sub–100-μm thickness, (B) intimate fit to the substrate, and (C) high optical translucency. (**D** to **F**) Trueness assessment by RMS deviation of the intaglio surface relative to the CAD reference: (D) PμSL and (E) CNC-fabricated veneers shown as 3D deviation color maps. (F) Statistical comparison of RMS deviations confirms the higher manufacturing accuracy of PμSL. (**G** to **I**) MG evaluation: (G) Schematic defining MG. Graphics were created by Y.S. using PowerPoint. (H) Representative optical images used for MG measurement and (I) quantified MG values showing smaller gaps for PμSL veneers within the ideal clinically acceptable ranges. (**J** to **L**) Compressive fracture testing: (J) schematic of the loading configuration, (K) representative specimens, and (L) load-displacement curves. The fracture load of an ∼80-μm PμSL zirconia veneer is not significantly different from that of a ∼500-μm milled glass-ceramic veneer, indicating sufficient strength for clinical handling.

In addition, an advantageous balance between translucency and color-masking ability was achieved (Fig. 5C). In clinical esthetics, veneer must transmit light naturally while still masking underlying discoloration. Lithium disilicate glass-ceramic is widely used for veneers because of its favorable translucency, but its combination of high light transmission and lower flexural strength often necessitates increased thickness to both conceal color and withstand functional loads. Zirconia has historically been limited in anterior esthetic by its lower translucency, motivating approaches that increase yttria content and the fraction of optically isotropic cubic phase (c-phase) to enhance translucency (*45*, *46*). However, increasing c-phase reduces the transformation-toughening contribution of tetragonal zirconia, typically compromising strength (*47*). Because translucency generally increases as thickness decreases (*20*, *21*), ultrathin geometries provide an alternative route that allows 3YSZ to achieve clinically acceptable optical outcomes without sacrificing its intrinsic mechanical advantage. After sintering, the veneers can be further shaded and glazed to achieve biomimetic color matching (fig. S10).

Manufacturing accuracy was quantified by three-dimensional (3D) surface deviation analysis between the CAD design and scanned intaglio surfaces. Representative deviation maps and root mean square (RMS) value are shown in Fig. 5 (D and E). Regions within 20 μm are shown in green. Yellow and red indicate outward (positive) deviation, whereas cyan and blue indicate inward (negative) deviation. Both groups showed unimodal deviation distributions, but the PμSL veneer exhibited a distribution centred near zero with a more symmetric spread and a larger fraction of low-deviation regions. No pronounced dark-blue or dark-red areas were observed, indicating the absence of severe local concavity/convexity. In contrast, the milled veneers showed a slightly shifted peak (∼0.02 mm) and more frequent high-deviation regions. Pronounced concave deviations were commonly observed near margins—consistent with the edge defects seen by microscopy—whereas convex deviations tended to appear in regions of rapidly changing curvature, such as corners of the intaglio surface. Because the intaglio surface forms the bonding interface, convex deviations can create localized “fulcrum” contacts that prevent full seating and compromise fit. Statistical comparison confirmed that PμSL ultrathin veneers achieved an RMS deviation of 20.4 ± 1.3 μm, smaller than milled regular-thickness veneers (31.8 ± 2.2 μm) in this study and lower than values reported for other additively manufactured veneers (*48*), supporting high trueness and process stability (Fig. 5F).

Marginal fit was evaluated because the veneer margin defines the boundary between the prepared (or minimally prepared) tooth and the oral environment (Fig. 5G). Marginal fit commonly quantified by the marginal gap (MG) (*49*). Inadequate marginal adaptation can promote ingress of microorganisms, saliva, and pigments, increasing the risks of secondary caries, adhesive degradation, and marginal staining; it can also elevate stress concentration at the restoration edge, raising the likelihood of ceramic chipping and fracture and reducing clinical longevity. Clinically, marginal adaptation may be judged qualitatively by probing and visual criteria (for example, USPHS-style ratings). For model-based evaluation, a widely used approach is the silicone replicate technique combined with microscopy and image analysis. Here, we used the silicone replica method (Fig. 5H), which captures the combined effects of intraoral scanning error, manufacturing accuracy, and seating behavior. With a designed cement space of 20 μm, the MG of PμSL ultrathin veneers was 20.71 (17.75) μm, whereas that of milled regular-thickness veneers was 50.58 (47.71) μm (Fig. 5I). A systematic review reported mean MGs for anterior porcelain laminate veneers spanning 22 to 315 μm, with more than 65% of studies in the range 70 to 158 μm (*49*). These comparisons suggest that the PμSL ultrathin veneers provide superior marginal adaptation, highlighting the value of high-precision ceramic 3D printing for producing consistent, well-defined margins that may benefit long-term restoration durability.

Last, we evaluated the handling-related strength relevant to clinical try-in and cementation, where finger pressure during seating is a common source of veneer failure. Because ultrathin and conventional veneers differ substantially in thickness and geometry, standardized strength tests on uniform specimens do not fully capture clinical loading scenarios. We therefore developed a veneer-level compression test to emulate seating pressure (Fig. 5J and movie S2). Under identical testing, the ultimate load of the 80-μm PμSL veneer (31.2 ± 2.6 N) was not statistically different from that of a 500-μm milled veneer (34.9 ± 4.7 N) (Fig. 5, K and L). Together, these results demonstrate that the ∼80-μm PμSL veneer is not simply a thinner analog of conventional veneers but a restoration enabled by a distinct manufacturing regime that delivers higher trueness, improved marginal adaptation, and clinically relevant handling strength at a fraction of the thickness. Further gains in performance are expected through continued refinement of slurry formulation, exposure and build strategies, postprocessing, and structural design, motivating future studies on durability and longer-term clinical outcomes.

### Clinical application of no-preparation veneers

Ultrathin veneers offer a minimally invasive restorative option that can reduce or potentially eliminate tooth preparation, thereby simplifying clinical workflows while preserving enamel and supporting long-term oral health (Fig. 6A and fig. S11) (*50*–*53*). Compared with CNC milling, high-precision ceramic 3D printing enables fabrication knife-edge margins with high geometric accuracy, reducing the need for manual grinding. At the tooth-veneer transition, the ultrathin veneer can integrate smoothly with the native tooth surface, supporting esthetics and patient comfort while maintaining a favorable marginal profile.

**Fig. 6.**
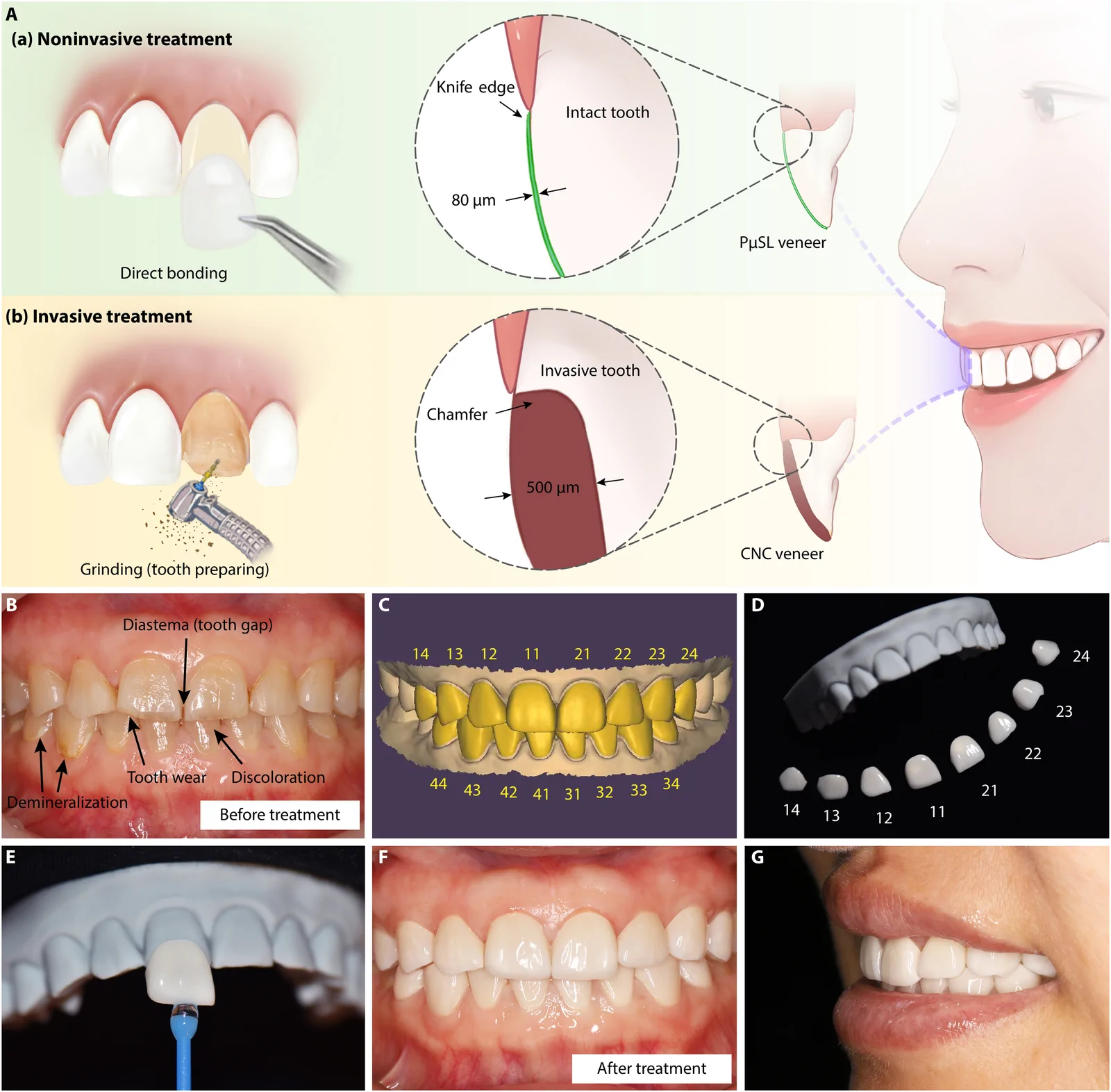
Proof-of-concept clinical application of PμSL ultrathin zirconia veneers. (**A**) Conceptual comparison of treatment approaches: no-/minimal-preparation restoration using an ∼80-μm PμSL-printed veneer (top) versus a conventional, more invasive approach using an ∼500-μm CNC-milled veneer (bottom). Graphics were created by S. Chen using Procreate. (**B** to **G**) Representative case treated with ultrathin veneers. (B) Pretreatment condition featuring diastema (gap between teeth), discoloration (caused by demineralization, aging, etc.) and wear (caused by chewing, brushing teeth, etc.). (C) CAD design of the veneers. (D) Fabricated PμSL veneers before bonding. (E) Extraoral (in vitro) try-in and fit evaluation. (F) Intraoral delivery/placement of the veneers. (G) Posttreatment photographs demonstrating improved color and tooth form with closure of interdental spacing. The ultrathin veneers as a protective barrier against mechanical and chemical challenges, supporting stabilization of the worn enamel surface.

We are currently conducting a clinical study (ChiCTR2600116240, www.chictr.org.cn) of this treatment with a sample size of 50 teeth; this article presents one typical case. Before treatment, the patient exhibited a noticeable triangular diastema between the maxillary central incisors, together with chalky white spots lesions consistent with demineralization and transverse grooves indicative of mechanical wear (Fig. 6B). In the digital workflow, the dentition was captured using an intraoral scanner, and ultrathin veneers were designed for the anterior teeth and first premolars in both the maxillary and mandibular arches (Fig. 6C). Zirconia veneers were fabricated using the optimized PμSL process (Fig. 6D), and their fit, accuracy, and appearance were first examined on an in vitro dental model (Fig. 6E). The restorations were then bonded without tooth preparation using a clinically established adhesive protocol (*13*), resulting in immediate closure of the interdental spacing and improvement in tooth color and form (Fig. 6, F and G). Beyond esthetics, the zirconia veneer—owing to its high strength and biocompatibility—serves as a durable external barrier that may help shield underlying enamel from mechanical challenge, chemical attack, and biofilm-related stimuli.

Therefore, the clinical case presented here should be interpreted as a proof-of-concept demonstration of translational feasibility. Larger clinical cohorts and continued longitudinal follow-up will be required to further establish the long-term clinical performance of these ultrathin zirconia veneers.

## DISCUSSION

This study establishes oxygen-mediated PμSL as a strategy for manufacturing ceramic structures whose critical dimensions approach the scale at which scattering-induced overcuring and release-related damage become dominant. The central finding is that an oxygen-permeable interface can do more than reduce adhesion. In a high-solid-loading zirconia slurry, the projected optical field is broadened by particle scattering into a high-dose core and a low-dose lateral tail (*54*, *55*). Oxygen supplied through the permeable release film preferentially suppresses polymerization in this weakly illuminated tail, while the projected core remains sufficiently reactive to overcome inhibition and form a bonded layer (*30*). The result is an anisotropic curing response: Lateral overcuring is strongly reduced, but curing depth is largely preserved. This behavior shifts ceramic PμSL from a scattering-dominated regime to a compensation-accessible precision regime.

This mechanism should be distinguished from both classical CLIP and conventional ceramic stereolithography. In CLIP of homogeneous resins, the oxygen-permeable window primarily creates a liquid dead zone that prevents adhesion and enables continuous replenishment. In ceramic stereolithography, particle scattering is usually treated as an optical penalty that broadens features and reduces penetration depth. In the present system, these two phenomena interact. Multiple scattering creates a laterally extended photon field, and oxygen inhibition converts that field into a chemically filtered boundary. The cross-sectional contour data and reduced optical-threshold model further suggest that the inhibition zone is not a flat geometric layer. Unlike the overall optical attenuation caused by light absorbers uniformly dispersed in the ceramic slurry, the oxygen suppression region is spatially nonuniform: thinner above the high-dose projected core and thicker near the lateral scattering region. This explains why the AF interface suppresses edge growth without producing a proportional loss of cured depth and provides a complementary mode of regulation.

In addition, this study addresses the challenge of not only projecting an optical pattern of ∼80 μm but also reliably fabricating a centimeter-scale ceramic object containing ∼80-μm local features while maintaining lateral accuracy, sufficient curing depth, and structural integrity throughout multilayer printing. Therefore, beyond the scattering-selective inhibition mechanism, the oxygen-permeable release interface also provides two process-level advantages that are important for fragile ceramic thin-wall printing. First, the oxygen-inhibited interfacial region creates a liquid-like separation layer between the cured ceramic polymer composite and the projection window. Although the separation force was not directly measured in this study, the improved build outcomes are consistent with reduced interfacial adhesion and a lower risk of release-related damage. This effect is particularly important for ultrathin shell geometries, where interlayer cohesion and green-body strength are limited and even modest release stresses can initiate delamination or fracture (*29*). Second, in the immersed-film configuration used here, the flexible release film participates in the separation–recoating cycle through elastic deformation and recovery. Slurry replenishment is therefore assisted by film rebound and self-leveling rather than by a rigid blade sweeping across fragile newly printed features. This recoating mode can help form uniform thin layers and reduce recoating-related defects, including leading-edge accumulation, thereby supporting interlayer continuity, *z*-axis fidelity and surface quality (*56*). These two process-level effects act synergistically with oxygen-mediated threshold filtering, providing a strategy for high-precision fabrication of extreme-scale components where build orientation and support placement are highly constrained.

At the same time, we acknowledge that certain limitations remain. The regulation of oxygen flux is one of the key limitations in this field and represents a direction for future research. In this study, we compared two release interfaces with markedly different oxygen permeability but did not independently control oxygen partial pressure, oxygen flux, or dead-zone thickness. From a mechanistic standpoint, oxygen flux is expected to affect the effective inhibition strength, dead-zone thickness, release behavior, lateral curing boundary, and final defect formation. This relationship is likely to be especially complex in high-solid-loading ceramic suspensions, where oxygen transport occurs through a particle-filled resin phase with reduced accessible diffusion volume and increased tortuosity. Future studies could use release films with graded oxygen permeability, controlled external oxygen partial pressure, oxygen-sensitive probes, and coupled reaction-diffusion models to determine how oxygen flux shifts lateral accuracy, curing depth, release force, and defect formation in ceramic PμSL.

Throughput, cost, and scalability also require further development. For ceramic vat photopolymerization, the manufacturing cycle is limited not only by optical exposure but also by postprocessing, especially debinding and sintering. Conventional ceramic parts require slow binder removal to avoid internal pressure buildup, cracking, or bloating. In contrast, the ∼80-μm local wall thickness of the present veneers shortens the diffusion path for binder decomposition products, allowing debinding to be reduced to several hours and providing an efficiency advantage over thicker ceramic components. At the current projection area of 19.2 mm × 10.8 mm, a single build can accommodate approximately five to six veneers with a build time of about 8 hours. Further scale-up will require larger or stitched projection fields, improved irradiance uniformity, optimized recoating programs, stable slurry leveling, and careful control of sedimentation and thermal gradients during debinding and sintering. The cost structure will depend mainly on AF film lifetime, printer throughput, thermal processing, and quality-control tolerance, but these costs may decrease as the process matures.

Esthetic translation and geometry-specific design rules represent important next steps. At present, the esthetic performance of zirconia restorations can be adjusted by selecting zirconia compositions with different yttria contents and translucency levels, and by postsintering staining, glazing, polishing, or sintering-related optical control. These approaches are practical and compatible with current dental workflows, but they mainly modify the restoration after the geometry has already been formed. A more advanced direction would be to integrate oxygen-mediated PμSL with multivat, multimaterial, or graded-composition printing, so that zirconia composition, pigment concentration, translucency, or even mechanical reinforcement could be spatially varied within a single veneer. Such intrinsic gradients may better reproduce enamel–dentin-like optical transitions and further expand the design space for minimally invasive esthetic restorations.

The strategy is not limited to the current zirconia slurry, but each material system will require recalibration. Higher solid loading, larger particle size, or greater refractive index mismatch would be expected to strengthen the scattering tail and increase lateral overcuring, whereas changes in photoinitiator system, absorber content, resin formulation, and release-film oxygen permeability would shift the effective curing threshold and apparent inhibition length. Future material diversification will also affect geometric design rules. For graded or multimaterial veneers, local optical properties, shrinkage behavior, interlayer bonding, and green-body stiffness may vary spatially, making geometry-process interactions more complex. Quantitative design rules, such as minimum radius of curvature, minimum unsupported span, maximum curvature gradient, allowable support density, and region-specific compensation limits, would therefore be valuable. Their systematic derivation will require geometry libraries, controlled printing trials, in situ or postprint defect mapping, and validated mechanical simulations that link local geometry, curing history, support strategy, and postsintering deformation. These studies will be essential for translating oxygen-mediated PμSL from proof-of-concept ultrathin veneers to broadly customizable esthetic ceramic restorations.

Despite these limitations, the present work shows that the anisotropic coupling between optical scattering and chemical threshold control can reformulate the long-standing trade-off between lateral resolution and curing depth in ceramic photopolymerization. The future studies outlined above will help move oxygen-mediated PμSL beyond its initial clinical demonstration in ultrathin veneers, toward deeper mechanistic understanding, more systematic theoretical development, and broader applications in ceramic microfabrication.

## MATERIALS AND METHODS

### Zirconia slurry and PμSL system

A commercial zirconia photopolymer slurry (BMF-PZSAMV-2, BMF Tech (Zhuhai) Inc., China) was used in the study. The slurry contained zirconia powder, multifunctional acrylate monomer, photoinitiator, dispersant, and solvent. The solid content is ∼80 wt %, and the viscosity is 12,892 ± 51 mP·s at the shear rate of 50 s^−1^ (fig. S12A).

Ceramic green bodies were fabricated using a PμSL printer (microArch, BMF Precision Tech Inc., China) with a nominal pixel size of 10 μm (fig. S12B). The printer used a 405-nm light-emitting diode light source and a digital micromirror device to project patterned light through the release film into the zirconia slurry. The available light-intensity range was 0 to 100 mW/cm^2^. The output intensity was calibrated using a digital optical power and energy meter (PM100D, Thorlabs, USA), and the uniformity of the projected light field in the *xy* plane was verified before the curing experiments (fig. S12, C and D).

Two commercial fluoropolymer release films with different oxygen permeabilities were used to regulate interfacial curing. A PFA film (Aorita, China) was used as the oxygen-impermeable control film, whereas an AF film (Teflon AF, Chemours, USA) was used as the oxygen-permeable film. The oxygen permeability of the AF film obtained from manufacturer was 990 barrer.

The release film was fixed at a projection-window/slurry interface, which remains submerged in the slurry during the printing process. After each projection, the curing part is peeled off from the release film, moved down with the printing platform, and then up again; the distance between the top of the curing part and the release film is the printing layer thickness.

### Single-layer line curing under PFA and AF interfaces

Straight-line projections were used as simplified single-layer models of narrow sliced features arising from ultrathin veneer margins and curved thin-shell regions. To compare interfacial curing behavior between release films, 80-μm-wide line patterns were exposed under PFA and AF films at a fixed intensity of 50 mW/cm^2^. Exposure times ranged from 2 to 20 s, corresponding to exposure doses of 100 to 1000 mJ/cm^2^. After exposure, cured ceramic-polymer composite specimens were removed from the slurry and cleaned to eliminate uncured slurry from the surface. Three-dimensional surface profiles were measured using a laser scanning microscope (VK-X200, Keyence, Japan) and a white-light interferometer (S neox, Sensofar, Spain).

For each cured line, cured width (W) was defined as the lateral maximum width of the solidified feature, and cured depth ($C_{d}$) was defined as the maximum vertical height/depth of the cured profile relative to the film-contact/reference plane. Excess width was calculated as: $W_{\text{ex}}=W-W_{d}$, where $W_{d}$ is the design width. For AF/PFA comparison at the same exposure dose, the reduction in excess width ($R_{w}$) and reduction in cured depth ($R_{d}$) were calculated as

$$R_{w}=\frac{W_{\text{ex},\text{PFA}}-W_{\text{ex},\text{AF}}}{W_{\text{ex},\text{PFA}}}\times 100\%$$ (8)

$$R_{d}=\frac{C_{d,\text{PFA}}-C_{d,\text{AF}}}{C_{d,\text{PFA}}}\times 100\%$$ (9)

### Optimization of stereolithography process parameter

To determine whether exposure energy alone could describe microscale curing in zirconia slurry, line patterns with different designed widths were fabricated under AF-mediated curing. The design width ranged from 20 to 200 μm. To decouple the effects of light intensity and exposure time under nominally identical total energy, three intensity-time combinations with the same exposure dose of 500 mJ/cm^2^ were compared: 25 mW/cm^2^ for 20 s, 50 mW/cm^2^ for 10 s, and 75 mW/cm^2^ for 6.7 s. Cured width, excess width, and cured depth were quantified using the same profile-measurement procedure described above.

Thin-wall arrays were printed to translate the single-layer curing behavior into multilayer structures. The models consisted of parallel thin sheets with designed wall thicknesses corresponding to the design width ranged from 20 to 200 μm, a nominal height of 1 mm, and a nominal length of 5 mm. Multilayer printing was performed under AF-mediated curing while varying layer thickness and exposure time. Layer thicknesses of 10, 20, and 30 μm were tested. Exposure time was varied across the selected range shown in Fig. 3F at an intensity of 50 mW/cm^2^. After printing, green bodies were separated from the build platform and cleaned using the same postprinting procedure described above. Build success was defined as formation of an intact thin-wall structure that remained attached during printing and survived postprinting cleaning without collapse, detachment, or gross fracture. Failed conditions were marked as gray regions in the process-window map.

To directly assess the influence of photocuring parameters on structural integrity before sintering, green-body flexural specimens were printed under selected exposure conditions. The nominal specimen dimensions were 20 mm in length, 4 mm in width, and 1.2 mm in thickness. The printing and loading directions correspond to the *x*, *y*, and *z* axes shown in fig. S6. Three-point bending strength was tested using a universal mechanical testing machine (Instron 3367, Instron, USA), with a support span of 16 mm and a crosshead speed of 1 mm/min. Flexural strength was calculated as: σ=3FL/(2bh2), where F is the maximum fracture load, L is the support span, b is the specimen width, and h is the specimen thickness.

### Thermal treatment and characterization

Zirconia samples were printed using optimized stereolithography process parameters, and thermogravimetric analysis and thermal mechanical analysis were performed to determine the debinding and sintering program. Dimensional shrinkage was quantified by measuring the *x*-, *y*-, and *z*-direction dimensions of specimens before and after sintering. Linear shrinkage was calculated as: $L=\left(L_{\text{green}}-L_{\text{sintered}}\right)/L_{\text{green}}\times 100\%$, where $L_{\text{green}}$ and $L_{\text{sintered}}$ are the corresponding dimensions before and after sintering.

The sintered surface and cross section were examined by field-emission scanning electron microscopy (SU-8010, HITACHI, Japan). Crystalline phases were analyzed by x-ray diffraction (Smartlab, Rigaku, Japan) with the scanning step of 0.02°, and the scanning range 2θ angle of 10° to 70°. Accelerated hydrothermal aging was performed in high-pressure steam at 134°C for 5 hours. The monoclinic phase fraction was estimated from the local peak areas/intensities near the monoclinic and tetragonal reflections.

The surface treatment and bonding protocol are based on the literature (*13*). Half of the bonded specimens were thermocycled for 5000 cycles between 5° and 55°C before shear bond testing. Shear bond strength was calculated as: τ=F/A, where F is the maximum debonding load and A is the bonded area.

### Ultrathin dental veneer fabrication and in vitro evaluation

In a simulation of the clinical procedure, a standard maxillary dental model was scanned with an intraoral scanner (Aoralscan 3, Shining 3D, China). An ultrathin veneer with an average thickness of ∼80 μm was designed for the right maxillary central incisor. Between the labial surface of the tooth and the intaglio surface of the veneer, a 20-μm cement gap was reserved. The veneer geometry was exported as a Standard Tessellation Language file, named *C*_pμsl_. The veneer model was laid out with the long axis of the tooth perpendicular to the printing platform and processed before printing, including adding support structure, compensatory scaling, and slicing into a series of patterns. Then, the optimized parameters obtained from the previous experiments were used for printing, with a dwell time of 60 s, a release rate of 0.5 mm/s, and a release acceleration of 0.5 mm/s^2^. Subsequent to the printing process, the green body of the veneers was detached from the printing platform and subjected to a series of postprocessing steps, including ultrasonic cleaning, debinding, and sintering. After laser removal of the support structure, grinding or polishing operations were performed to smoothen veneer surfaces. According to the manufacturer’s recommendation, the lithium disilicate glass-ceramic (IPS e.max CAD, Ivoclar Vivadent, Liechtenstein) veneer with an average thickness of ∼500 μm was milled by a four-axis grinder (AIM-4Z, Aoke Technology, China), noted as *C*_mill_. The cement gap and intaglio surface of both veneers are the same.

The margin of the veneers was observed and graded under a microscope at high magnification. An industrial 3D scanner (GOM ATOS Q, Zeiss, Germany) was used to scan the veneers, and the data were saved as *S*_pμsl_ and *S*_mill_. 3D surface deviation analysis between scan data and CAD data was performed to evaluate the processing accuracy of the veneer. The RMS value is a quantitative metric that directly reflects the overall deviation level between the test model and the reference model and is generally used to evaluate the manufacturing accuracy. In this experiment, a negative correlation was observed between the RMS value and the processing accuracy of the veneer. The calculation formula is as follows (*57*, *58*)

$$\text{RMS}=\sqrt{\frac{1}{N}\sum_{i=1}^{N}d_{i}^{2}}$$ (10)

where $d_{i}$ is the single-point deviation value and *N* is the total number of points measured.

The marginal fit of the veneers was assessed using the silicone replica technique. Light-body silicone (Virtual light body, Ivoclar Vivadent, Liechtenstein) was mixed and applied to the intaglio surface of the veneer. Similar to the actual cementation process, the veneer was positioned on the tooth model, and a constant pressure of 500*g* was applied on the labial surface until the silicone polymerized. After polymerization, excess material was removed from the margins and the veneer was carefully separated, leaving the light-body silicone membrane adhered to the tooth model. Then heavy-body silicone (Virtual heavy body, Ivoclar Vivadent, Liechtenstein) was applied over the light-body silicone membrane to create the silicone replica. The silicone replica was removed from the model and sectioned in the mesial-distal and incisal-gingival directions. The MG, which refers to the gap between the veneer margin and the tooth labial surface, was measured under microscope.

In addition, a universal mechanical testing machine was used to perform a compressive test on the veneer. Silicone columns placed on the labial surface of the veneer transmitted compressive load to the veneer, approximating the distributed finger pressure used to seat restorations. The columns were fabricated using molds to control contact position and area, and the tooth base was designed with a hollow top to avoid unintended support during loading. The test was conducted at a rate of 1 mm/min until the veneer fractured. The load at fracture was named as the ultimate load (newtons).

### Statistical analysis

Normally distributed quantitative data are presented as means ± SD, whereas non-normally distributed data are presented as median and interquartile range. Statistical analyses were performed using the statistical software (SPSS Statistics 27, IBM Corp., USA). Normality and homogeneity of variance were assessed using Shapiro-Wilk test and Levene’s test. For two-group comparisons, an independent-samples *t* test was used when the data met parametric assumptions; otherwise, the Mann-Whitney *U* test was used. For multiple-group comparisons, one-way or two-way analysis of variance (ANOVA) followed by Tukey post hoc analysis was used when appropriate. Statistical significance was set at *P* < 0.05.
