# Supplementary material for: Oxygen-mediated projection microstereolithography for sub–100-μm zirconia dental veneers
Source: Sci Adv. 2026 Nov 4;12(45):eaef9661. doi: 10.1126/sciadv.aef9661 (PMC13733828; doi:10.1126/sciadv.aef9661)
Supplement: Supplementary file 1 [file sciadv.aef9661_sm.pdf]

Science Advances
AAAS

Supplementary Materials for

Oxygen-mediated projection microstereolithography for sub–100-μm zirconia dental veneers

Yanru Shen *et al.*

Corresponding author: Xiang Wang, xiangwang@pku.edu.cn; Nicholas X. Fang, nicxfang@hku.hk; Yuchun Sun, kqsyc@bjmu.edu.cn

Sci. Adv. **12**, eaef9661 (2026)
DOI: 10.1126/sciadv.aef9661

The PDF file includes:

Figs. S1 to S12
Tables S1 and S2
Legends for movies S1 and S2

Other Supplementary Material for this manuscript includes the following:

Movies S1 and S2

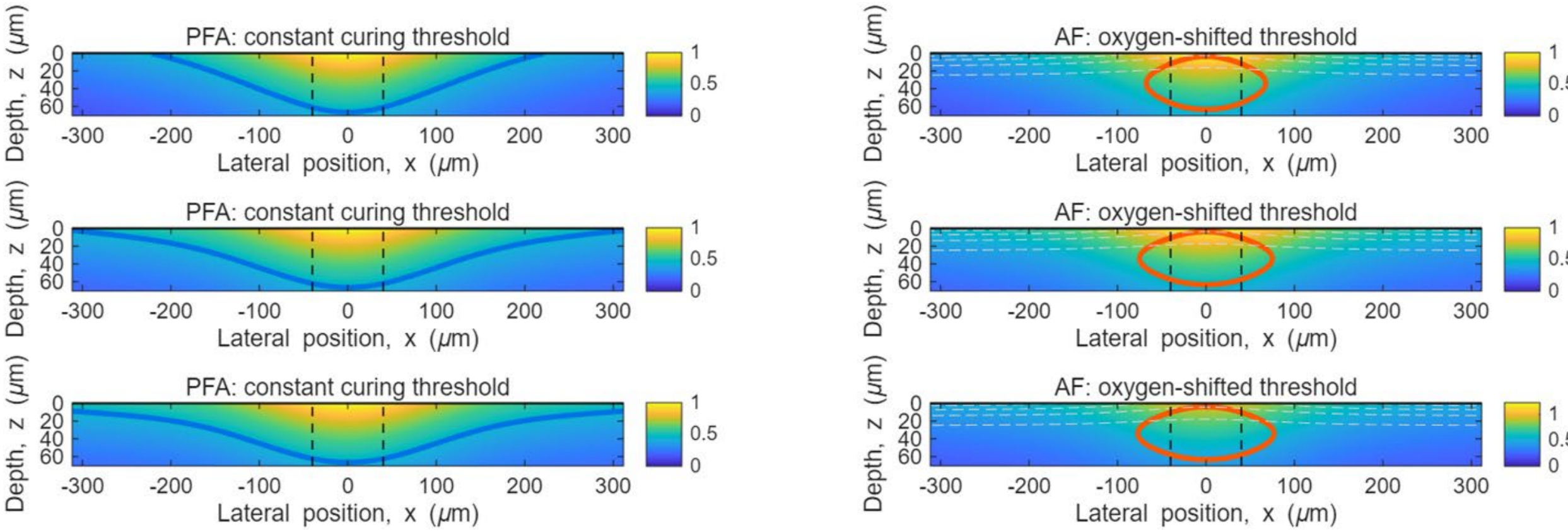

Fig. S1. Effect of lateral scattering length. Curing contour of PFA group (left) and AF group (right) with different $\boldsymbol{L_s}$.

Increasing the lateral scattering length L_s from 400 μm to 1200 μm increased the maximum PFA cured width from 440 μm to 868 μm, whereas the AF cured width changed only modestly from 132 μm to 152 μm. This indicates that the broad PFA overcured skirt is primarily controlled by the lateral scattering tail, while the AF oxygen-shifted threshold suppresses much of this scattering-induced lateral expansion.

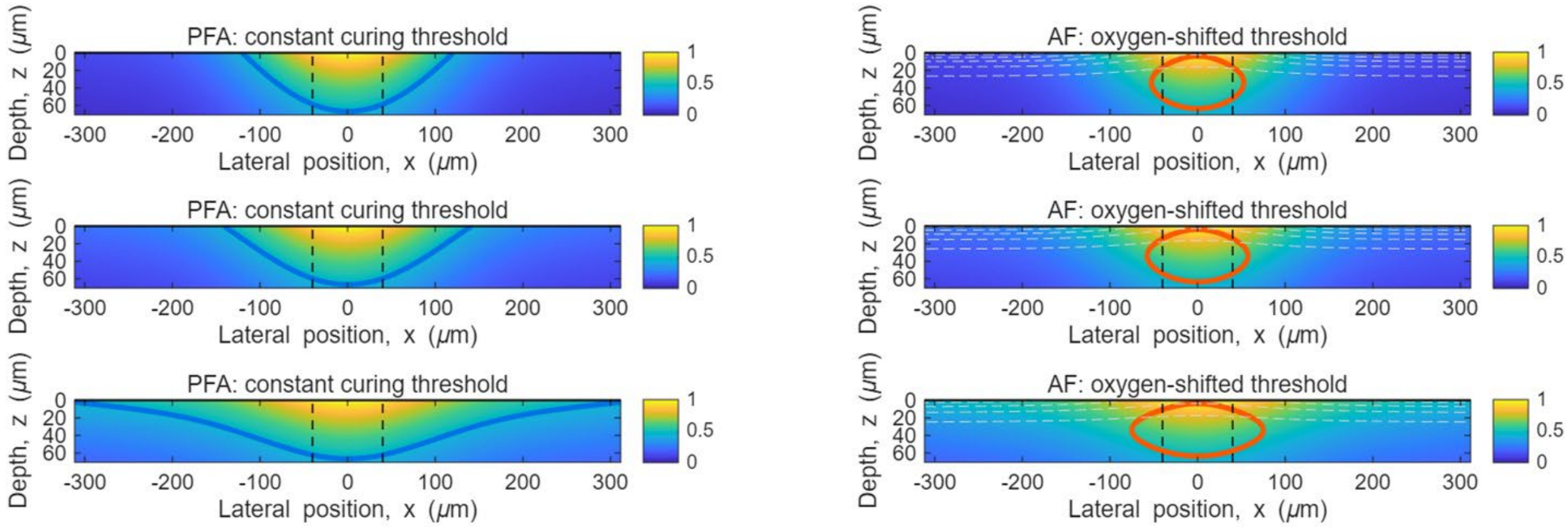

Fig. S2. Effect of scattering-tail. Curing contour of PFA group (left) and AF group (right) with different $\boldsymbol{\beta}$.

A similar trend was observed when increasing the long-tail weighting factor β. As β increased from 0.1 to 0.9, the maximum PFA cured width increased from 212 μm to 660 μm. The AF width also increased, but remained much smaller than the PFA width. This supports the role of the scattering-tail component in generating lateral overcuring under the oxygen-impermeable interface.

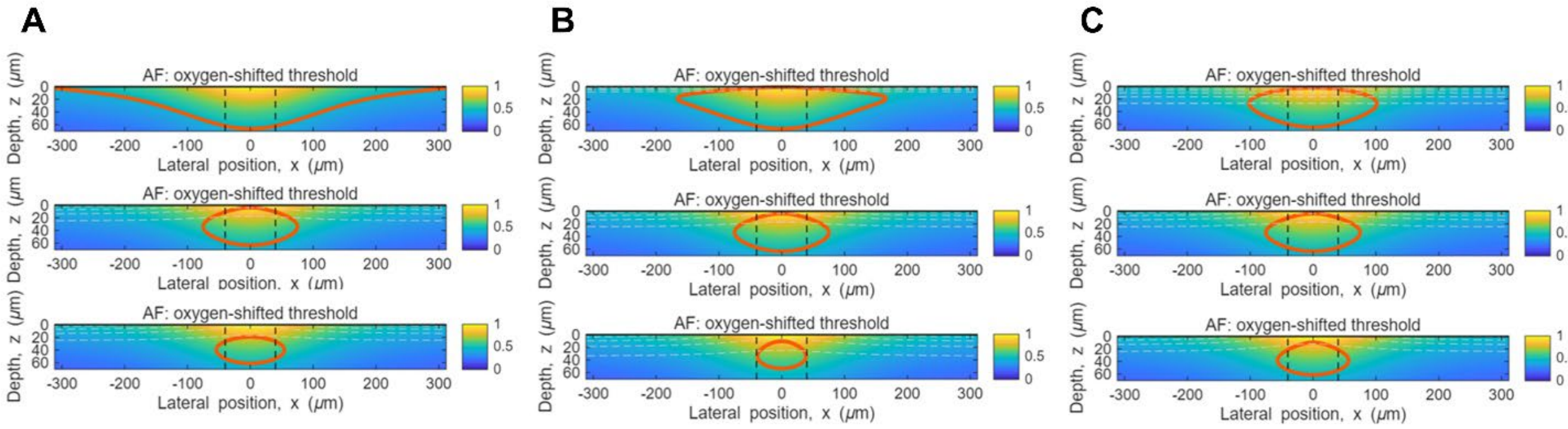

Fig. S3. Effect of oxygen-inhibition parameters. Curing contour of AF group with different (A) $\boldsymbol{A}$, (B) $\boldsymbol{\lambda}$, and (C) $\boldsymbol{\eta}$.

The oxygen-inhibition strength A controlled the transition from a film-attached to a film-detached AF contour (**Fig. S3A**). When $A = 0$, the AF contour was identical to the PFA contour, with a maximum width of 660 μm and no interfacial dead zone. Increasing A to 0.6 reduced the AF width to 148 μm and generated a nonzero dead zone. Further increasing A to 0.9 narrowed the AF contour to 108 μm, but also reduced the cured thickness from 59 μm to 41 μm. These results show that oxygen inhibition is required to detach and narrow the AF contour, but excessive inhibition can compromise cured thickness.

The apparent inhibition length λ regulated how deeply oxygen inhibition extended into the slurry (**Fig. S3B**). Increasing λ from 5 μm to 20 μm reduced the AF width from 332 μm to 76 μm and reduced the maximum cured thickness from 65 μm to 42 μm, while increasing the center dead-zone thickness from 1 μm to 11 μm. Thus, a shallow interfacial inhibition length allows lateral overcuring to be suppressed while preserving sufficient cured thickness, whereas an overly deep inhibition field excessively suppresses polymerization.

The dose-selective inhibition coefficient η was essential for reproducing the nonuniform dead zone observed experimentally (**Fig. S3C**). When $\eta = 0$, the model produced a nearly uniform inhibition gap, with a small difference between the edge and center dead-zone thicknesses. The dead-zone nonuniformity increased with the increasing η. This result indicates that the low-dose scattering tail is more susceptible to oxygen inhibition than the central high-dose projection area.

Together, the sensitivity analysis supports the proposed oxygen-mediated anisotropic inhibition mechanism. Scattering-related parameters control the broad overcured skirt under PFA, oxygen inhibition parameters control detachment and narrowing under AF, and the dose-selective inhibition term is required to generate the nonuniform dead zone characteristic of AF curing.

Therefore, the simulated contour transition is not a result of a single arbitrary parameter set, but arises from the coupled effects of lateral scattering and oxygen-shifted curing thresholds.

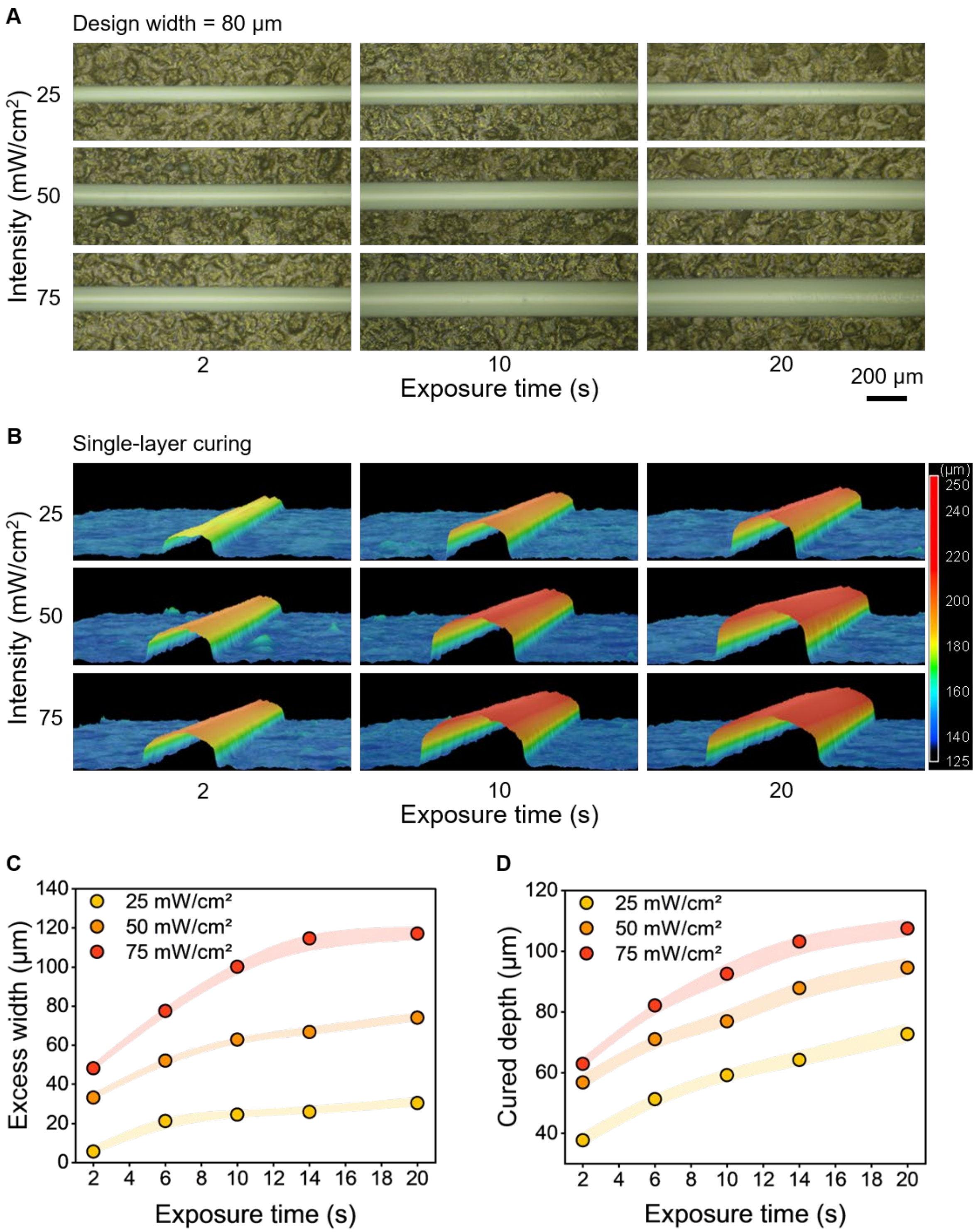

Fig. S4. Exposure parameter dependence of AF-mediated curing behavior. (A) Microscope images and **(B)** scanning profiles of the cured fine-line specimens with a design width of 80 μm

exposed at different parameters. **(C** to **D)** Influence of exposure dose for an 80 μm design line on (C) excess width and (D) cured depth.

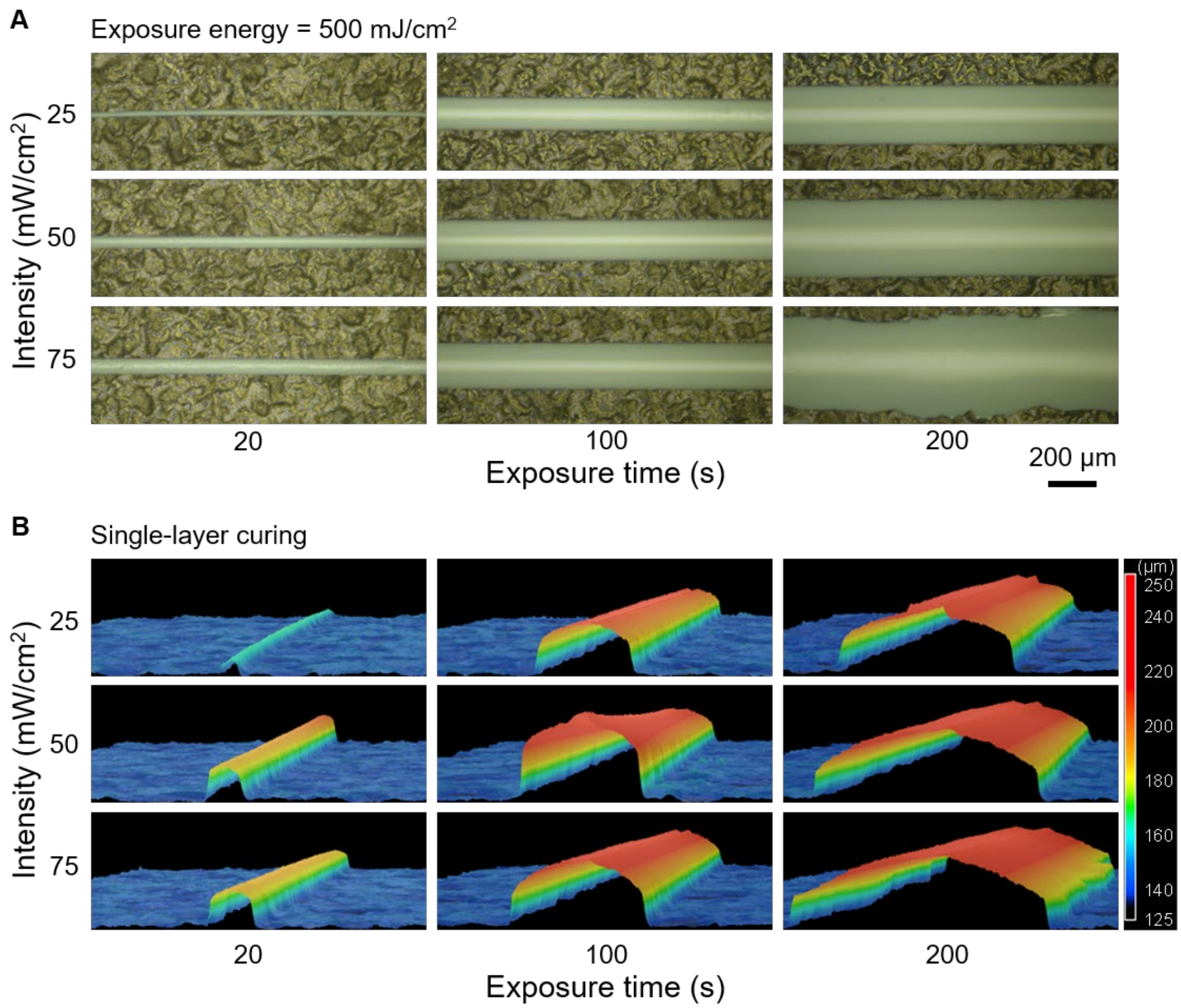

Fig. S5. Representative morphology of the cured fine-line specimens with different design widths exposed at 500 mJ/cm^2. (A) Microscope images. **(B)** Scanning profiles.

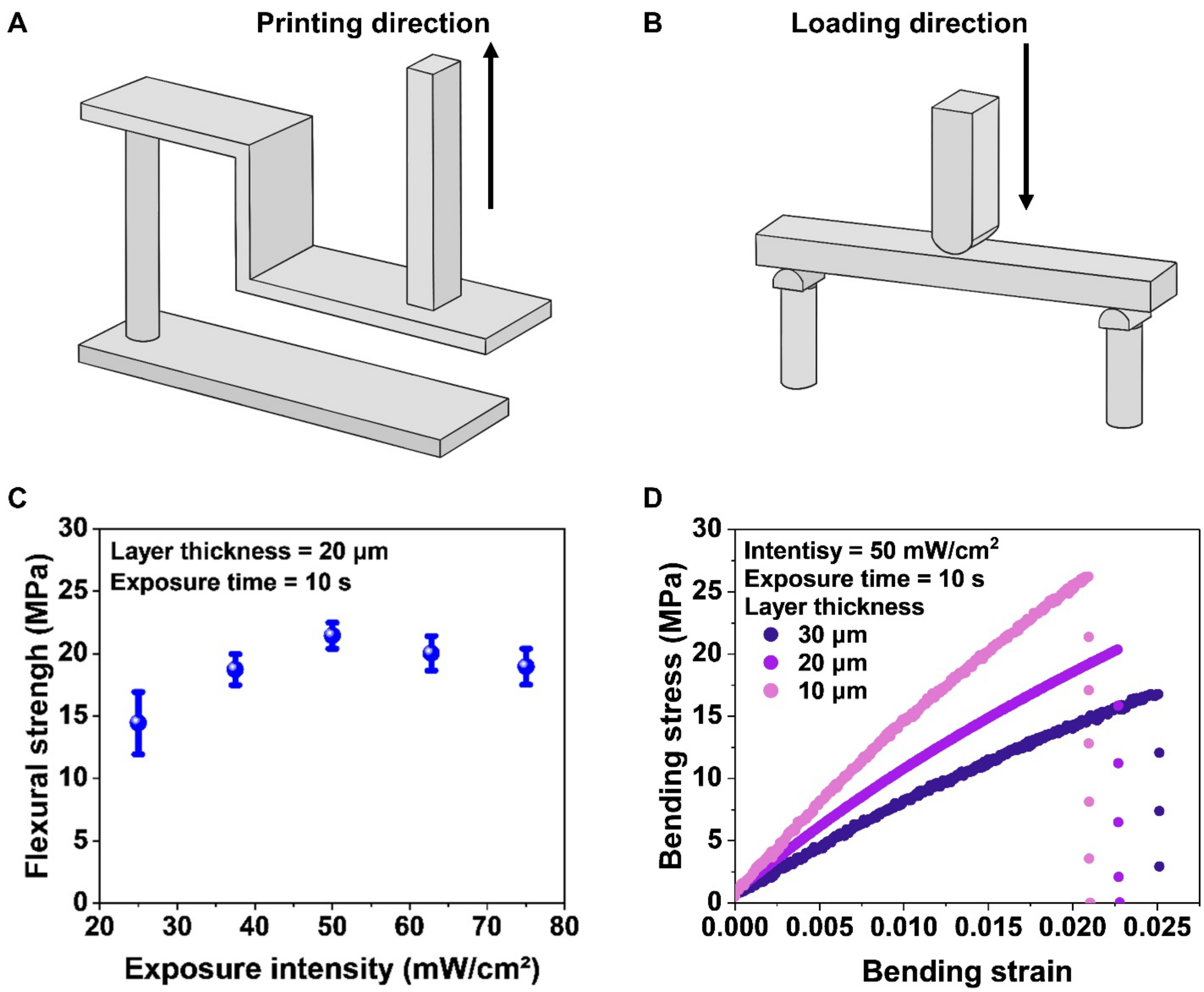

Fig. S6. Optimization of printing parameters and specimen orientation for mechanical evaluation. Schematic illustration of the specimen orientation during **(A)** printing and **(B)** three-point bending testing. Graphics were created by Shuqin Chen using Procreate. **(C)** Flexural testing of green bodies identified a moderate exposure intensity (50 mW/cm^2) that maximizes mechanical performance while maintaining printability. **(D)** Reducing the printing layer thickness improved strength.

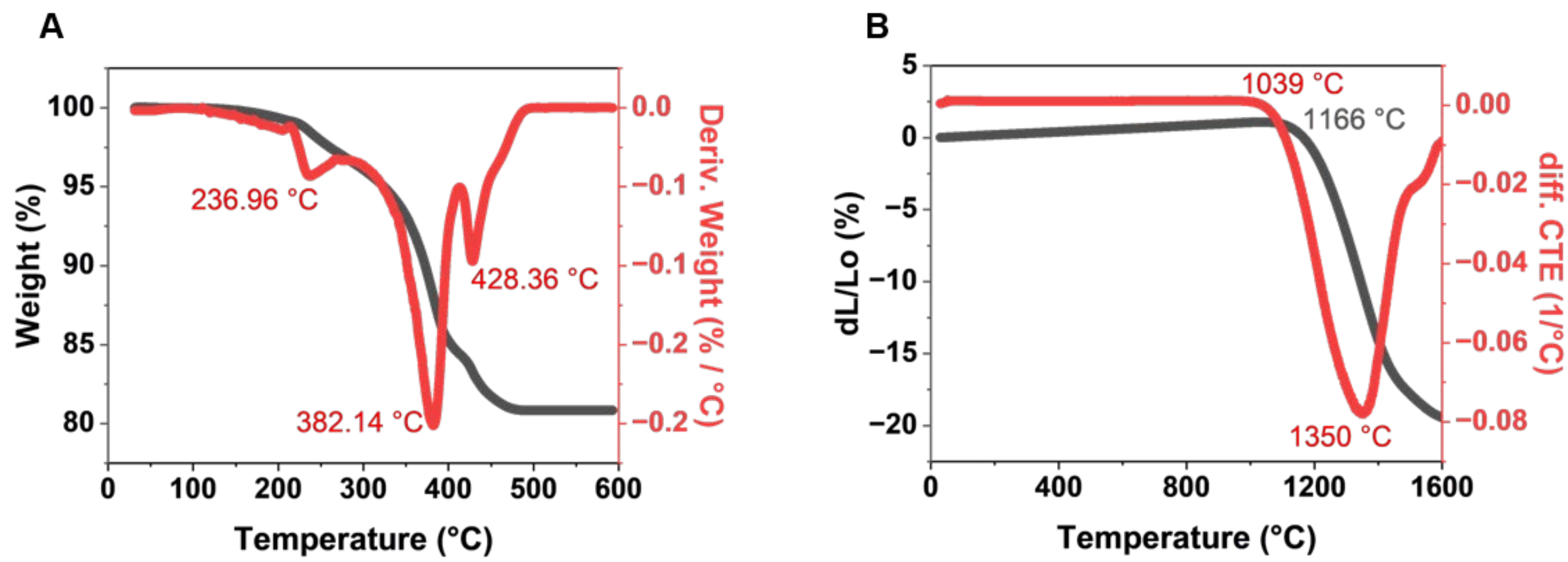

Fig. S7. Thermal analysis-guided debinding and sintering schedule. (A) Thermogravimetric (TG) analysis and **(B)** thermomechanical analysis (TMA) used to guide debinding and sintering schedule.

The TG curve approaches a plateau after ~500 °C, indicating that most organic constituents in the green body have decomposed below this temperature. The DTG curve exhibits three major mass-loss events at 236.9 °C, 382.1 °C, and 428.3 °C, allowing the debinding process to be divided into three stages. From room temperature to ~230 °C, low molecule-weight species volatilize. Between 230 °C and 380 °C the polymeric binder undergoes chain scission and decomposition into smaller hydrocarbons, accompanied by volatilization of products with higher saturated vapor pressure. From ~380 °C to ~430 °C, the remaining low-volatility decomposition products and residual carbon are removed; with further heating in air, residual carbon is oxidized to CO_2 and carried away. This TG-DTG guided staging informs the use of reduced heating rates across the principal decomposition windows to mitigate internal gas build-up and associated cracking or distortion of ultra-thin parts.

After debinding, densification proceeds during the high-temperature sintering. TMA reveals the onset of sintering at ~1039 °C, consistent with particle rearrangement and the initiation of neck formation. At ~1166 °C, shrinkage accelerates as grain boundaries develop and the microstructure consolidates, with the shrinkage rate peaking at ~1350 °C. Although the shrinkage rate decreases at higher temperatures, densification continues. Sintering at 1550 °C for 2h yield high density and favorable mechanical performance, whereas further temperature increases are expected to promote grain coarsening and can reduce mechanical strength.

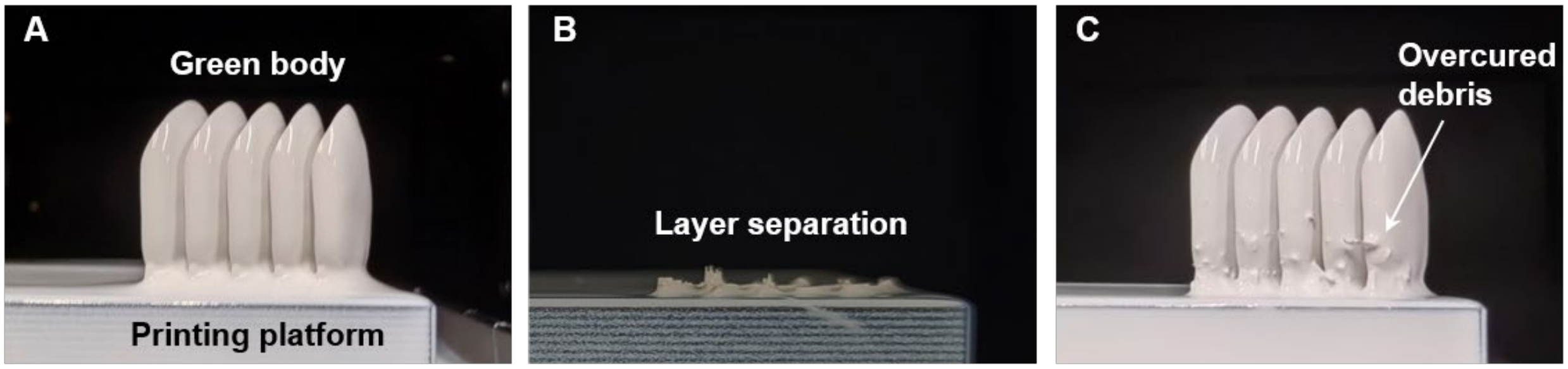

Fig. S8. Effect of release-film oxygen permeability and parameter optimization on ultra-thin veneer build reliability: (A) Optimized printing using the oxygen-permeable AF film produced intact ultra-thin zirconia veneer green bodies. **(B)** Printing with the oxygen-impermeable PFA film failed to retain an intact green body. **(C)** Using the AF film with non-optimized parameters resulted in irregular overcured debris.

Using the oxygen-permeable film together with the optimized printing parameters produced intact ultra-thin veneer green bodies on the build platform (**Fig. S8A**). In contrast, the oxygen-impermeable PFA film failed to retain a complete green body under otherwise matched conditions, suggesting that adhesion between the cured layer and the window exceeded the interlayer cohesion of the fragile veneer structure and led to layer separation during printing (**Fig. S8B**). When the oxygen-permeable film was used with non-optimized exposure parameters, veneer green bodies could be formed, but their surfaces contained irregular overcured debris, indicating that oxygen mediation alone was insufficient without an appropriate exposure and layer-thickness window (**Fig. S8C**).

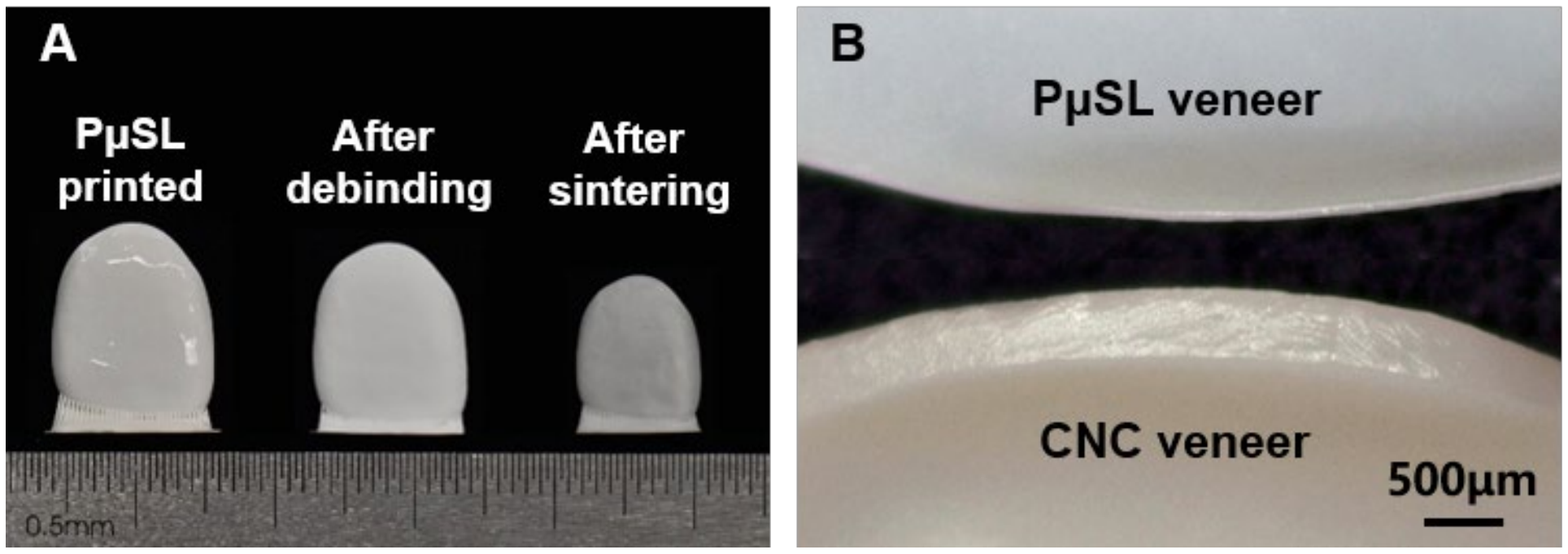

Fig. S9. Fabrication process and edge morphology of PμSL-fabricated veneers. (A) Different fabrication stages. **(B)** Thin and flawless edge of the PμSL-fabricated veneer compared to the CNC-milled veneer.

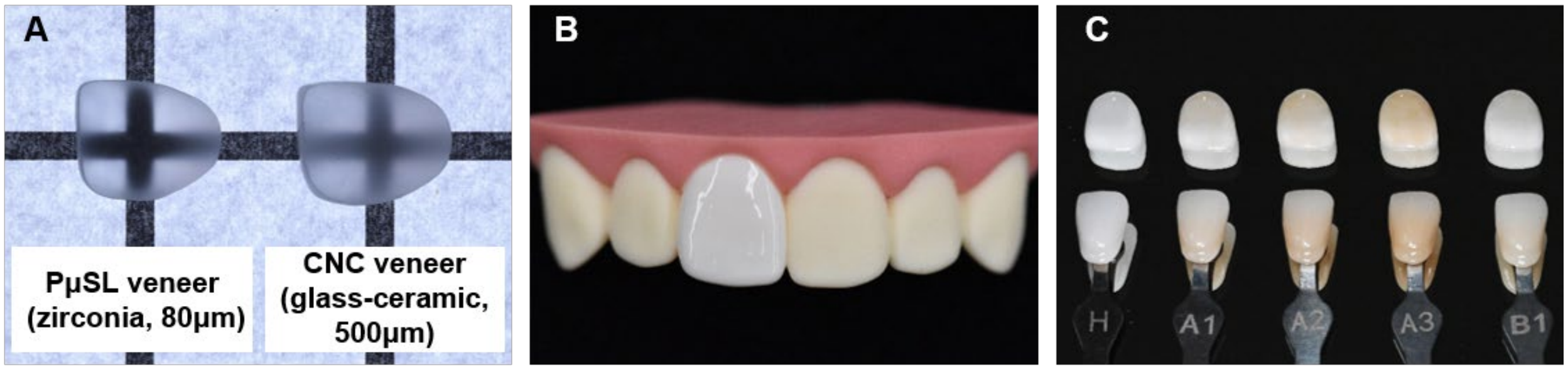

Fig. S10. Optical performance and shade-matching potential of ultrathin zirconia veneers. Perfect balance of **(A)** high translucency and **(B)** effective color-masking ability. **(C)** Various shades to mimic the tooth.

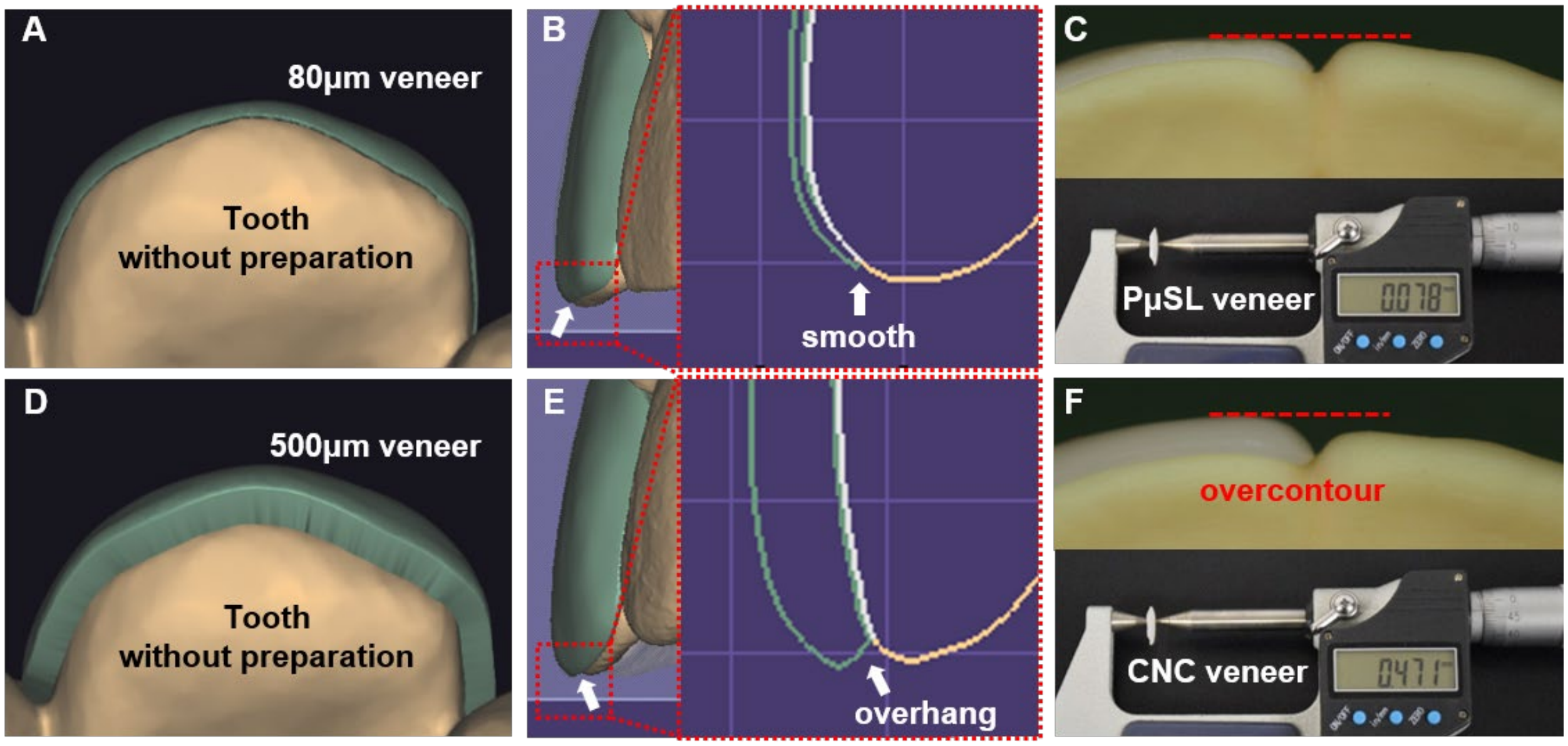

Fig. S11. Contour comparison of veneers with different thicknesses (A to C: 80μm, D to F: 500μm) placed on the unprepared teeth. (A, D) Lingual view. **(B, E)** Mesiodistal section. **(C, F)** Contour consistency with symmetrical teeth.

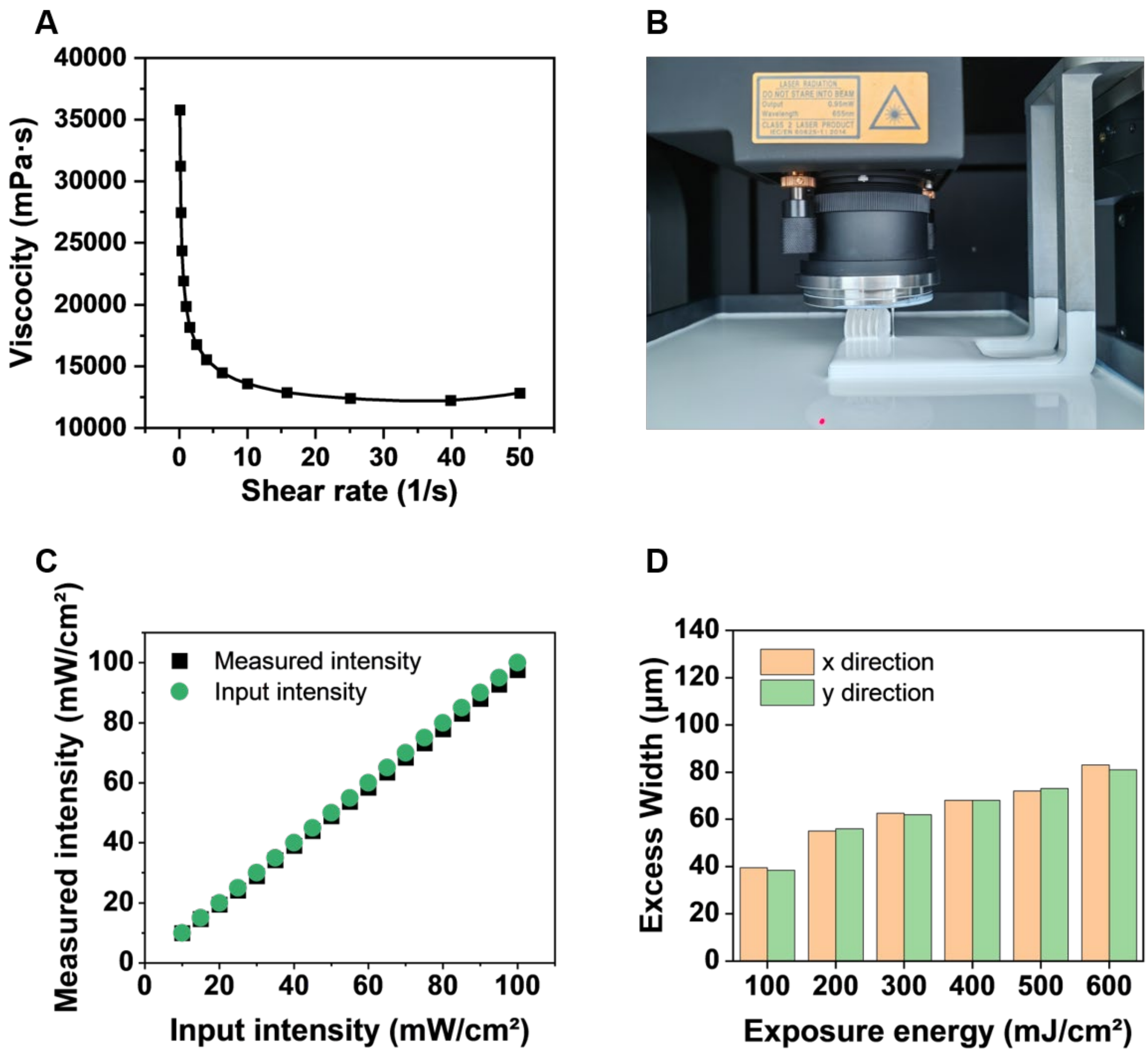

Fig. S12. Characterization and calibration of the PµSL printing system. (A) Viscosity as a function of shear rate. **(B)** Projection Micro Stereolithography system. **(C)** Consistency between input and output intensities. **(D)** Consistency of projected light in the x and y directions.

Table S1. Parameters in sensitivity analysis of optical-threshold contour model.

Parameter	Meaning	Baseline in Fig. 2H	Sensitivity range
σ	Effective near-interface broadening	80 μm	fixed
L_s	Effective scattering-tail length	800 μm	400–1200 μm
β	Weighting factor of scattering tail	0.9	0.1–0.9
μ	Effective attenuation coefficient	0.012 μm^{-1}	fixed
E_c	Normalized curing threshold	0.45	fixed
A	Effective oxygen-inhibition strength	0.6	0–0.9
λ	Apparent inhibition length	15 μm	5–20 μm
η	Dose-selective inhibition coefficient	1.8	0–3.6

We reproduced representative contour features (**Fig. 2H**) and performed parameter sensitivity analysis using the optical-threshold contour model (**fig. S1-S3**). These parameters were selected to and should be interpreted as normalized effective parameters rather than independently measured optical, oxygen-transport, or radical-kinetic constants.

Table S2. Monoclinic phase calculation.

Sample	Before aging	After aging
I_m(-111) area	7.98	45.97
I_t(101) area	1176.02	1248.93
I_m(111) area	3.83	3.44
X_m	0.99%	3.81%
V_m	1.30%	4.93%

Movie S1. Green bodies of ultra-thin veneer have been printed using the Projection Micro Stereolithography (PμSL) system.

Movie S2. Compression test process including silicone rubber deformation and veneer fracture**.**
